# Comparison of Depletion Strategies for the Enrichment of Low-Abundance Proteins in Urine

**DOI:** 10.1371/journal.pone.0133773

**Published:** 2015-07-24

**Authors:** Szymon Filip, Konstantinos Vougas, Jerome Zoidakis, Agnieszka Latosinska, William Mullen, Goce Spasovski, Harald Mischak, Antonia Vlahou, Joachim Jankowski

**Affiliations:** 1 Biomedical Research Foundation Academy of Athens, Biotechnology Division, Athens, Greece; 2 Charité–Universitätsmedizin Berlin, Berlin, Germany; 3 University of Glasgow Institute of Cardiovascular and Medical Sciences, Glasgow, United Kingdom; 4 Ss. Cyril and Methodius University in Skopje, Nephrology Department, Skopje, Former Yugoslav Republic of Macedonia; 5 Mosaiques Diagnostics GmbH, Hannover, Germany; 6 University Hospital RWTH Aachen, Institute for Molecular Cardiovascular Research, Aachen, Germany; UGent / VIB, BELGIUM

## Abstract

Proteome analysis of complex biological samples for biomarker identification remains challenging, among others due to the extended range of protein concentrations. High-abundance proteins like albumin or IgG of plasma and urine, may interfere with the detection of potential disease biomarkers. Currently, several options are available for the depletion of abundant proteins in plasma. However, the applicability of these methods in urine has not been thoroughly investigated. In this study, we compared different, commercially available immunodepletion and ion-exchange based approaches on urine samples from both healthy subjects and CKD patients, for their reproducibility and efficiency in protein depletion. A starting urine volume of 500 μL was used to simulate conditions of a multi-institutional biomarker discovery study. All depletion approaches showed satisfactory reproducibility (n=5) in protein identification as well as protein abundance. Comparison of the depletion efficiency between the unfractionated and fractionated samples and the different depletion strategies, showed efficient depletion in all cases, with the exception of the ion-exchange kit. The depletion efficiency was found slightly higher in normal than in CKD samples and normal samples yielded more protein identifications than CKD samples when using both initial as well as corresponding depleted fractions. Along these lines, decrease in the amount of albumin and other targets as applicable, following depletion, was observed. Nevertheless, these depletion strategies did not yield a higher number of identifications in neither the urine from normal nor CKD patients. Collectively, when analyzing urine in the context of CKD biomarker identification, no added value of depletion strategies can be observed and analysis of unfractionated starting urine appears to be preferable.

## Introduction

Advances in mass spectrometry (MS) have recently facilitated the development of high-throughput and sensitive analysis methods for proteomics investigations [1–3]. However, proteome analysis of complex biological samples remains challenging, among others due to the huge abundance differences among individual protein components; for example, in plasma, the presence of albumin or immunoglobulins (IgG) and other predominant proteins hinder the detection of less abundant proteins and reduces the efficiency of LC-MS/MS analysis [4]. This masking effect is also expected to be pronounced in the analysis of the urinary proteome of patients with chronic kidney disease (CKD) who present high levels of urinary albumin [5]. Furthermore, albumin abundance is highly variable between patients with CKD, even with the same disease etiology, which further complicates the analysis and comparison of the urinary protein content of these samples [6, 7]. Similarly to plasma [8], the range of protein concentration in urine spans several orders of magnitude [9, 10]. Due to the fact that the concentration of potential disease biomarkers might be relatively low, predominant proteins may mask them and make their identification challenging. Therefore, fractionation and depletion strategies are generally employed prior to MS analysis [11].

Currently, several fractionation methods for protein depletion are available. Some of them are based on the separation of proteins by physicochemical properties such as charge (ion-exchange [12]) or size (size-exclusion chromatography [13]), while others target specific protein groups or ligands, such as glycosyl groups in the case of glycoproteins [14] or biochemical properties (i.e. immunoaffinity [15]). These affinity chromatography methods are applicable for a rapid and selective depletion or enrichment of biomolecules from complex samples [16, 17]. The selection of a fractionation strategy depends on the specific study requirements. For example, combinatorial peptide ligand libraries, allow for the simultaneous depletion of highly-abundant proteins and enrichment of low-abundance targets, facilitating their detection by MS [18]. However, this approach requires relatively high amounts of starting material (hundreds of milliliters of urine) to ensure efficient enrichment of low-abundance proteins; otherwise, high- and medium-abundance proteins would not fully saturate their ligands and ultimately the elution would have the same profile as initial sample [19–21]. Since in most cases low volumes of urine (<1 mL) are available when investigating prospectively collected samples from clinical cohorts, combinatorial ligand peptide libraries do not appear to be applicable for analysis of such individual urine samples. [21]. Strategies based on the depletion of abundant proteins require lower initial material compared to combinatorial peptide ligand libraries [21, 22]. These strategies include immuno-based depletion methods involving selective binding of target proteins to the stationary phase based on affinity. They are considered to have high specificity and efficiency and achieve rapid purification or concentration of the analytes [15]. Another depletion strategy is based on ion-exchange chromatography relying on attraction of oppositely charged molecules as the basis for separation [12].

Depletion of abundant proteins appears especially relevant when investigating the urinary proteome of CKD patients, where the levels and variability of highly-abundant proteins noticeably increase with each stage of CKD [5]. On the other hand, depletion of abundant proteins causes co-depletion of several low-abundance proteins, hindering their detection [23–25]. Several protein depletion kits are commercially available. These kits are generally designated to be used for plasma samples and their application has been evaluated in several manuscripts (e.g. [22, 24, 26–28]). Kulloli et al. [28] applied a kit for depletion of 14 abundant proteins in plasma prior to analysis by LC-MS/MS. The depletion allowed to enrich the sample for low-abundance proteins and increased the number of identifications compared to the non-depleted sample (from approx. 71 to 130 proteins). Similarly, Tu et al. [26] observed a 25% increase in the number of identifications when kits depleting 7 or 14 high-abundance proteins were applied prior to the LC-MS/MS analysis. However, the authors questioned the applicability of the depletion strategy for the identification of disease biomarkers in plasma, since the low-abundance proteins accounted only for 6% of total identifications and 50 of the proteins with the highest abundance accounted for 90% of total spectral counts. Along the same lines, two-dimensional gel electrophoresis (2DE) analyses of plasma samples, where depletion of abundant proteins strategy was applied, demonstrated an increase in the number of spots on the gel. Yet, most of the newly identified spots, represented different isoforms of high-abundance proteins (e.g. albumin, IgGs) [24, 27].

Various protein depletion kits have been also tested on urine samples [29–32]. Afkarian et al. [31] depleted albumin and IgG from urine of diabetic patients with or without nephropathy. Subsequently, iTRAQ labeling was performed and the samples were analyzed by 2D-LC-MS (MALDI-TOF/TOF). No increase in the number of identified proteins was observed in the depleted samples, regardless if the patient was normo- or macro-albuminuric. On the other hand, Kushnir et al. [30] reported a 2.5-fold increase in the number of protein identifications by LC-MS/MS after depleting 6 highly abundant proteins (albumin, IgG, alpha-1 antitrypsin, IgA, transferring and haptoglobin) using multiple affinity removal (MARS) column (Agilent Technologies, Santa Clara, CA). Abundant protein depletion strategies (14 MARS) in conjunction with iTRAQ labeling were also applied for the identification of potential bladder cancer biomarkers from urine [33]. The depletion strategy allowed increasing the number of identifications from approximately 300 proteins in the non-fractionated sample to 500, and the discovery of a potential biomarker panel for bladder cancer [33].

Collectively, based on the existing conflicting data it is presently unclear whether depletion strategies are of benefit when analyzing urine samples. In this study, we therefore aimed to assess the effectiveness of different commercially available depletion strategies for the proteome analysis of urine samples from CKD patients and healthy controls: four different strategies (three immunodepletion- and one ion-exchange-based) were applied prior to LC-MS/MS analysis. The efficiency of depletion, reproducibility, and the overall impact of each strategy on the number of protein identifications and relative protein quantification were assessed.

## Materials and Methods

### Sample characteristics

Second morning mid-stream urine samples were employed. To remove cell debris, urine was centrifuged at 1,000xg for 10 min at 4°C. Two pooled urine samples (with a final volume of approx. 30 mL each) corresponding, to normal and CKD (stage IV) were generated. Protein content was estimated by Bradford protein assay. To reduce freeze-thaw cycles to minimum, samples were aliquoted in 500 μL (40 aliquots per CKD and normal pool) and kept at -20°C until used. Sample collection was performed in accordance to local ethics requirements and the study was approved by the local ethics committee ("Macedonia Academy of Sciences and Arts"; ethics subcommittee for medicine, pharmacy, veterinary and stomatology: 07–65711, 1-04-2013). All individuals gave written informed consent.

### Chromatography approaches

500 μL urine aliquots (corresponding to a protein content of 29 μg for normal and 437 μg for CKD sample) were subjected to buffer exchange applying buffers compatible with each depletion method according to the respective manufacturer, and concentrated to a final volume of 20 μL, using Amicon Ultra Centrifugal Filter Units (3kDa cut-off, Millipore).

Such prepared samples were processed with four commercially available kits targeting the depletion of abundant proteins (Table 1) according to the manufacturers' protocols. To assess the reproducibility of each method, five technical replicates of each of the urine samples from healthy controls and from CKD patients per technique were prepared. Depleted samples were obtained either from the flow-through fraction for three immuno-based kits: Seppro IgY14 (Sigma-Aldrich, Saint Louis, MO, USA), ProteoPrep (Sigma-Aldrich, Saint Louis, MO, USA) and SpinTrap (GE Healthcare, Little Chalfont, UK) or in the elution fraction for the ion-exchange kit: ProteoSpin (Norgen Biotek, Thorold, Canada). Protein content after depletion was quantified by Bradford protein assay. The protocol for each depletion kit is briefly described below:

**Table 1:** Characteristics of the applied depletion strategies.

Depletion kit	Company	Mechanism	Depleted proteins
**Seppro IgY14**	Sigma Aldrich	Immunodepletion	Albumin, IgG, α1-Antitrypsin, IgA, IgM, Transferrin, Haptoglobin, α2-Macroglobulin, Fibrinogen, Complement C3, α1-Acid Glycoprotein (Orosomucoid), HDL (Apolipoproteins A-I and A-II), LDL (mainly Apolipoprotein B)
**ProteoPrep**	Sigma Aldrich	Immunodepletion	Albumin, IgG
**SpinTrap**	GE Healthcare	Immunodepletion	Albumin, IgG
**ProteoSpin**	Norgen Biotek	Ion-exchange	Albumin, alpha-1-antitrypsin, transferrin and haptoglobin

Seppro IgY14: (loading capacity: up to 1000 μg of total protein content) After buffer exchange to “Dilution Buffer” (100 mM Tris-Buffered Saline, Tris-HCl with 1.5 M NaCl, pH 7.4) and concentration to 20 μL, urine sample was further diluted with the “Dilution Buffer” to a final volume of 500 μL. Depletion column was centrifuged to remove the storage buffer and the sample was applied to the column. In brief, the sample was thoroughly mixed with the column resin and incubated on an end-to-end rotator for 15 minutes. This step ensures binding of target proteins to the resin. Afterwards, the sample was centrifuged and the first depleted fraction was collected. Subsequently, to increase the recovery rate of proteins not binding to the resin, 500 μL of “Dilution Buffer” was added onto the column and centrifuged once more. Two fractions (0.5 mL each), corresponding to depleted sample, were combined prior to filter-aided sample preparation (FASP) for LC-MS/MS analysis. The depleted sample was analyzed by SDS-PAGE and LC-MS/MS. To prepare the column for another use, bound proteins were stripped off the column resin by applying “Elution Buffer” (1 M glycine, pH 2.5) followed by 3 min incubation, according to the manufacturer’s instructions. Afterwards, the column resin was rinsed and kept in the storage buffer until further use.

ProteoPrep: (loading capacity: up to 3000 μg of total protein content) After buffer-exchange to “Equilibration Buffer” (low ionic strength Tris buffer, pH 7.4) and concentration to 20 μL, the sample was further diluted with “Equilibration Buffer” to a final volume of 100 μL. Diluted sample was then loaded onto the equilibrated column (prepared according to the manufacturer’s instructions) and incubated for 10 minutes to allow binding of the target proteins to the column resin. This step was repeated once. The sample was centrifuged and in order to collect remaining unbound proteins, 125 μL of “Equilibration Buffer” was added onto the column. The depleted sample comprised of the flow-through from previous step and the wash (in total 225 μL). The depleted sample was analyzed by both SDS-PAGE and LC-MS/MS. To collect bound proteins for analysis by SDS-PAGE, the column was eluted twice with 150 μL of “Protein Extraction Reagent” (40 mM Trizma Base, 7.0 M urea, 2.0 M thiourea and 1% C_7_BzO detergent, pH 10.4). Elution fraction was also kept for further analysis by SDS-PAGE.

SpinTrap: (loading capacity: up to 3000 μg of total protein content) After buffer-exchange to “Binding Buffer” (20 mM sodium phosphate, 0.15 M sodium chloride, pH 7.4) and concentration to 20 μL, the sample was diluted with the Binding Buffer to a final volume of 100 μL. The column was equilibrated, the sample was applied onto the column and incubated for 5 min. Unbound sample components were collected by centrifugation, and the column was washed twice with 100 μL of “Binding Buffer”. The depleted sample comprised of these three collected fractions (flow-through of the loaded sample and two washes—300 μL) and was further analyzed by SDS-PAGE and LC-MS/MS. Bound proteins were eluted by adding 150 μL of “Elution Buffer” (0.1 M glycin-HCl, pH 2.7) twice. These obtained fractions (300 μL) were also combined and further analyzed by SDS-PAGE.

ProteoSpin: (loading capacity: up to 500 μg of total protein content) After buffer-exchange to “Binding Buffer” (20 mM sodium phosphate, 0.15 M sodium chloride, pH 7.4) and concentration to 20 μL, the sample was diluted with the “Column Activation and Wash Buffer” (composition not specified by the manufacturer) to a final volume of 500 μL. The column was activated followed by application of the diluted sample. During this step, the non-targeted proteins bind to the resin. Afterwards, the samples were centrifuged. The flow-through containing the highly-abundant target proteins was kept for SDS-PAGE analysis. The column was then washed twice with 500 μL of “Column Activation and Wash Buffer”. 100 μL of the “Elution Buffer” (composition not specified by the manufacturer) was added and the column was centrifuged. This step was repeated twice. Collected fractions (200 μL) were combined. This depleted sample was analyzed by SDS-PAGE and LC-MS/MS.

### 1-dimensional gel electrophoresis (SDS-PAGE)

15μL of each chromatography fraction were loaded on a 10% acrylamide gel and SDS-PAGE was performed. The gels were stained with silver [34].

### Sample preparation for LC-MS/MS

Urine samples (5 replicates each) prior to or after subjecting to fractionation (Table 1) were processed following the FASP protocol, commonly applied in our laboratory as described previously [35], with minor modifications. Specifically, in brief, samples were concentrated to a final volume of 50 μL using Amicon Ultra Centrifugal Filter Units (30kDa cut-off, Millipore, Billerica, MA, USA) at 13,000 rpm and incubated with 0.1 M 1,4-Dithioerythritol for 20 min. Subsequently, two centrifugal wash steps were performed by adding 200 μL urea buffer (8M urea in 0.1M TRIS-HCl, pH 8.5). After these centrifugation steps, protein alkylation was conducted by adding 100 μL of iodoacetamide solution (0.05M iodoacetamide in urea buffer) and incubating the mixture for 20 min in the dark. Afterwards, two additional washes with urea buffer were performed followed by two washes with ammonium bicarbonate (ABC) buffer (50mM NH_4_HCO_3_, pH 8). Overnight digestion was conducted by adding trypsin solution in ABC buffer (trypsin to protein ratio—1:100). Peptides were eluted by centrifugation followed by filter washing with 40 μL ABC solution. The peptide mixture was lyophilized and resuspended in 20 μL (for urine from healthy controls) and 200μL (for urine from CKD patients) of mobile phase A (0.1% formic acid), due to the different protein load of the two samples.

### LC-MS/MS analysis

6μL (corresponding to 30% for normal and 3% for CKD samples of the respective total peptide mixtures) of the prepared peptide mixture were analyzed on a Dionex Ultimate 3000 RSLS nano flow system (Dionex, Camberly UK). After loading onto a Dionex 0.1×20 mm 5 μm C18 nano trap column at a flow rate of 5 μl/min in 98% 0.1% formic acid and 2% acetonitrile, sample was eluted onto an Acclaim PepMap C18 nano column 75 μm×50 cm (Dionex, Sunnyvale, CA, USA), 2 μm 100 Å at a flow rate of 0.3 μl/min. The trap and nano flow column were maintained at 35°C. The samples were eluted with a gradient of solvent A: 0.1% formic acid; solvent B: 100% acetonitrile, 0.1% formic acid, starting at 2%B for 10 min, rising to 5%B at 11 min, 15%B at 73 min and 55%B at 95 min. The column was then washed and re-equilibrated prior to injection of the next sample.

The eluant was ionized using a Proxeon nano spray ESI source operating in positive ion mode into an Orbitrap Velos FTMS (Thermo Finnigan, Bremen, Germany). Ionization voltage was 2.2 kV and the capillary temperature was 250°C. The mass-spectrometer was operated in MS/MS mode scanning from 350 to 2,000 amu. The resolution of ions in MS1 was 60,000 and 15,000 for HCD MS2. The top 20 multiply charged ions were selected from each scan for MS/MS analysis using HCD at 35% collision energy.

### Protein identification and data processing

Protein identification was performed using the SEQUEST search engine (Proteome Discoverer 1.4, Thermo Scientific). Protein search was performed against the SwissProt human protein database (30.10.2013) containing 20277 entries without protein isoforms. The following search parameters were applied: i) fragment mass tolerance: 0.05Da; ii) full tryptic digestion; iii) max missed cleavage sites: 2; iv) static modifications: carbamidomethylation of cysteine; v) dynamic modifications: oxidation of methionine; vi) event detector mass precision: 2 ppm; vii) min. precursor mass: 600 Da; viii) max. precursor mass: 5000 Da; ix) min. collision energy: 0 eV; x) max. collision energy 100 eV; xi) target FDR (strict): 0.01; xii) target FDR (relaxed): 0.05; xiii) FDR validation based on: q-Value. Obtained results were further processed by applying the following filters: i) high confidence (FDR <1%); ii) mass peak deviation: 5 ppm; iii) at least one unique peptide per protein; iv) peptide and protein grouping were enabled. Additionally, since the same peptide can be associated with two (or more) different sequences in different experiments and hence be “lost” for comparison, we initially collected information on the top5 ranked sequences. In the next steps using an in-house developed software (described in the next paragraph), these sequences were harmonized so that the most probable sequence per peptide is assigned, improving the data consistency.

Specifically, the list of peptides was exported from “Proteome Discoverer” and processed further as follows; For each spectrum, the corresponding sequence was defined based on the relative number of sequence identifications in each sample. The relative quantitative analysis was performed based on the peptide area values. Obtained sequences for all technical replicates were merged. Peptides were assigned to the corresponding proteins after merging the list of peptides from 5 technical replicates. Peptides corresponding to multiple proteins were assigned to the protein identified based on the highest number of peptides (“Occam’s Razor rule” [36]). Due to a bug in “Proteome Discoverer”, for a limited number of peptide identifications the area was not retrieved. If such situation occurred, missing values were replaced by the mean area for the group. Only peptides reported in more than 60% of the samples (3 out of 5 technical replicates) were considered for the calculations of the number of peptide and protein identifications, protein peak areas, sequence coverage, evaluation of consistency and statistical analysis.

Protein peak area was calculated based on the average of top three most abundant peptides for a given protein. Subsequently, normalization of the protein peak areas was conducted. Depletion targets and putative targets were excluded from calculating total sample peak area, since levels of these proteins change between each method applied, introducing bias and falsely increasing the abundance of other proteins. Therefore, the data were normalized based on non-target proteins, which, in principle, should remain unchanged. The validity of this method was confirmed following a comparison of normalized values to ELISA measurements of albumin (data not shown). As putative depletion targets, we consider proteins with high homology to targeted proteins (Table 1), therefore of potential affinity to the corresponding antibody (for example, different complement factors—see S2 Table for the list of excluded proteins). Proteins identified with at least one unique peptide were included in the analysis.

Average protein area based on top 3 peptidesTotal peak area (of non−targets) in the sample based on average of top 3 peptides per protein*10^6

Immunoglobulin chains were combined into the following proteins, representing the abundant proteins from the group: Ig gamma-1 chain C region (comprising of lambda, gamma and kappa and heavy chains), Ig alpha-1 chain C region (comprising of Ig alpha chains and J chain) and Ig mu chain C region.

Statistical analysis was based on the unequal variance 2-tailed Student's t-test. Proteins with p-value ≤0.05 and ratio ≥1.5 or ≤0.66 were considered as statistically significant. Additionally, in the case of relative protein abundance, obtained p-values were adjusted by applying Benjamini-Hochberg correction for multiple testing.

## Results

### SDS-PAGE analysis

Four commercially available depletion kits were employed to estimate their efficiency and reproducibility in combination with LC-MS/MS analysis of urinary proteins. Five technical replicates were performed in each case, using urine from normal or CKD patients. In addition, 5 technical replicates of each of the urine from CKD and normal patients (unfractionated –starting material) were analyzed to assess effectiveness of protein depletion. Since the study aims at the evaluation of depletion strategies in biomarker discovery using samples from large clinical cohorts, where typically low-urine volumes are available per researcher, the analysis was performed using a starting volume of 500 μL (without targeting specific starting protein amounts, regularly not feasible in such studies).

**Fig 1:**
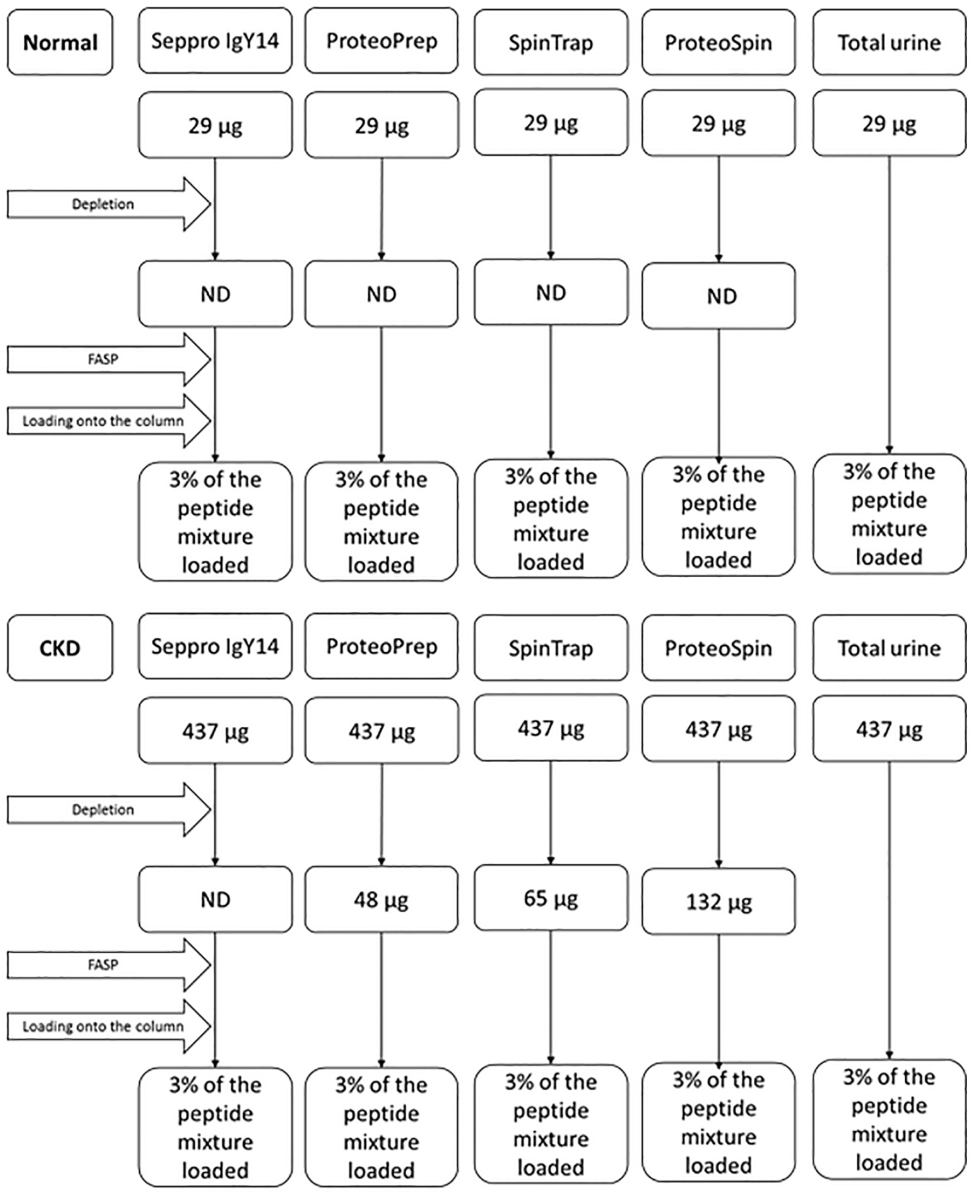
Protein amounts at different steps of the analysis as estimated by Bradford measurements. ND: not determined due to measurements being below the limit of detection (i.e. concentration < 0.2 μg/μL).

Fractionation was performed according to the manufacturer’s instructions with minor adaptations, as described in the Materials and Methods section. Bradford assay was performed to estimate the total protein content in urine samples after depletion. Protein amounts at different steps of the analysis, when determined, are presented in Fig 1. The total protein content prior to depletion was estimated at 29 μg (normal) and 437 μg (CKD). In the case of normal sample, the protein content after depletion was below the limit of detection, regardless of the method applied. For the CKD sample, after applying ProteoPrep and SpinTrap kits, the protein content was estimated at 48 μg and 65 μg respectively. The highest protein amount remaining in the sample after depletion was observed for ion-exchange-based ProteoSpin kit, (estimated at 135 μg). For Seppro IgY14, the respective protein content was below the limit of detection. As shown, protein measurements in the depleted fraction vary among different methods, as expected in part based on their specificity.

**Fig 2:**
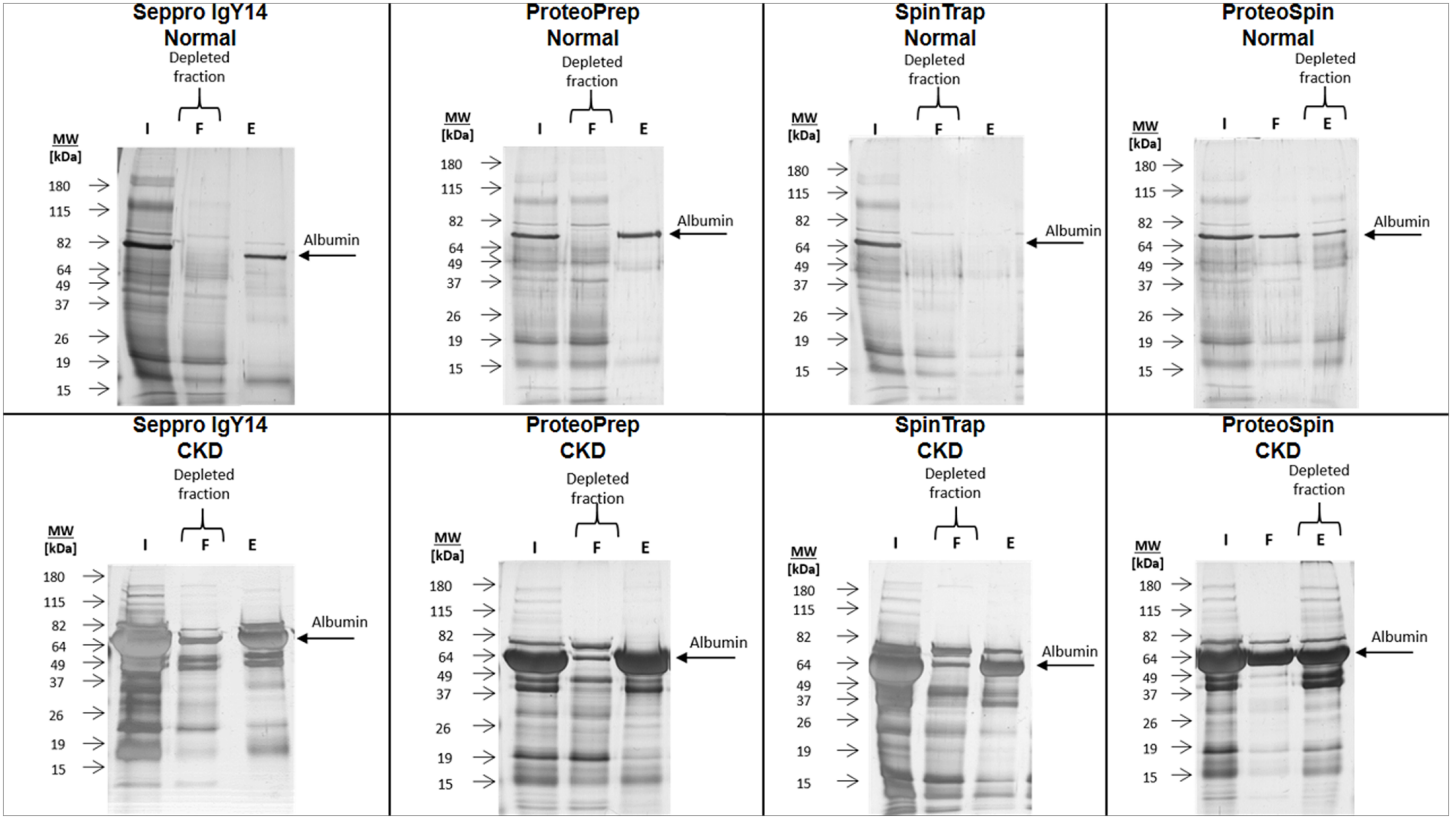
Representative SDS-PAGE results for fractionated and non-fractionated samples (normal and CKD). The figure represents initial urine, flow-through and elution for each of the depletion kits applied. The fractions representing depleted sample and albumin as a common protein depleted by all the kits are marked. I—Initial urine (non-fractionated sample); F—Flow-through fraction; E—Elution. The same protein amounts were loaded onto the gels for initial sample (lane 2 in all cases). Any observed differences in staining intensities are attributed to differences in the silver staining procedure.

Depleted urine fractions were then subjected to SDS-PAGE analysis to investigate efficiency and reproducibility of each depletion strategy. Representative gel fractions per method are presented in Fig 2 and all of the analyzed SDS-PAGE gels are shown in S1–S5 Figs. Gel patterns of the depleted fractions indicate reproducibility in all cases (evidenced in S1–S5 Figs), as estimated by their high similarity among technical replicates. As shown based on this gel image analysis, the immuno-based methods appear to have a higher depletion efficiency compared to the ion-exchange strategy, in overall agreement with the measured protein concentration.

### Urine peptides and proteins identified by LC-MS/MS

**Fig 3:**
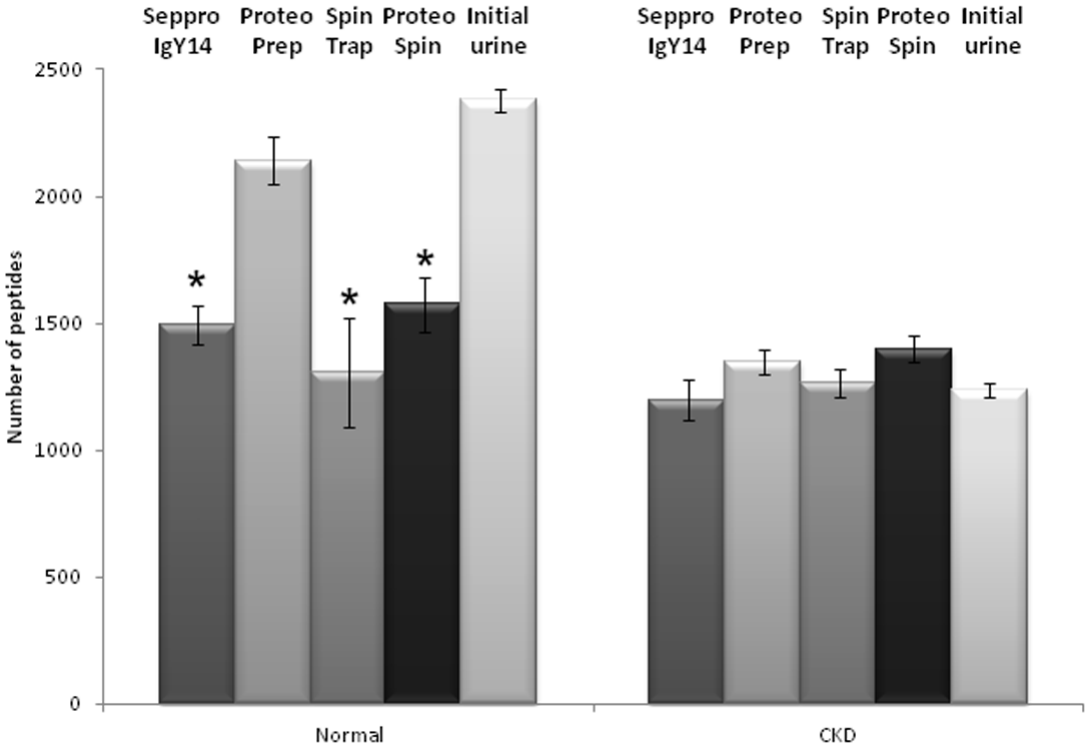
Average number of peptides identified per method.

Urine samples prior to or after depletion were processed according to the FASP protocol and analyzed by LC-MS/MS. The numbers of identified peptides per run for each of the five technical replicates per method were compared (Fig 3). For urine of healthy controls, the highest number of peptides was identified from the initial (unfractionated) sample (approx. 2,400 peptides) and in the depleted fraction processed by ProteoPrep kit (approx. 2,150 peptides), followed by ProteoSpin, Seppro IgY14 and SpinTrap kits (approx. 1,500 peptides). The most significant differences in the number of identifications, were found between initial urine and Seppro IgY14, SpinTrap and ProteoSpin kits (p-value ≤ 0.0002). In the case of urine from CKD patients, no significant difference in the number of detected peptides could be observed when comparing the output of the different methods (approx. 1250 peptides in the unfractionated and all depleted fractions).

To rule out that differences in the number of identifications is related to undersampling and/or MS data quality, we investigated the number of obtained peptides, number of PSMs, search inputs (MS/MS scans), and total ion currents (TICs) obtained in each case. As demonstrated in Table 2, the average numbers of PSMs, search inputs and TICs were comparable among CKD and normal samples per depletion strategy. Nevertheless, in the case of CKD, the number of peptide identifications is lower compared to the respective number from normal. This suggests that for CKD, a larger fraction of the MS/MS scans is on the same, highly-abundant peptides.

**Table 2:** Comparison of the number of peptide identifications, PSMs, search inputs and TICs for normal and CKD sample.

**Normal**				
Analysis method	Average number of identified peptides	Average number of PSMs	Average number of Search inputs	Average total ion current [sum of the peak areas]
Seppro IgY14	1495	4978	15813	7.98E+10
ProteoPrep	2142	6263	18092	2.55E+11
SpinTrap	1306	4363	15685	9.07E+10
ProteoSpin	1575	5184	15725	6.73E+10
Total urine	2380	10650	21576	3.86E+11
**CKD**				
Seppro IgY14	1197	5646	15905	5.06E+10
ProteoPrep	1350	6980	16628	9.02E+11
SpinTrap	1264	6667	16425	8.26E+10
ProteoSpin	1399	8772	19192	2.00E+11
Total urine	1234	9055	22455	4.34E+11

Comparable numbers of proteins identified in at least three out of five replicates per technique were detected in all cases (approx. 390 in normal and 160 in CKD samples). Overall, more proteins were detectable in the normal urine than in CKD sample (p-value = 0.0002). This observation applies for both total urine and fractionated samples (Table 3). All techniques were found to be reproducible in terms of received protein identifications, as shown in Table 3. In all cases, at least 80% of identified proteins were detected in all 5 replicates.

**Table 3:** Total number (sum) of identified proteins per depletion strategy for normal and CKD sample (in at least 3, 4 and 5 technical replicates). For both depleted and non-depleted sample the number of identifications is higher in normal than in CKD urine.

**Normal**					
**Name of the kit**	Seppro IgY14	ProteoPrep	SpinTrap	ProteoSpin	Total urine
**Proteins identified in 5 replicates**	287	387	265	276	362
**Proteins identified in 4 replicates**	321	420	299	315	397
**Proteins identified in 3 replicates**	354	466	352	361	431
**CKD**					
**Name of the kit**	Seppro IgY14	ProtoPrep	SpinTrap	ProteoSpin	Total urine
**Proteins identified in 5 replicates**	113	151	159	116	132
**Proteins identified in 4 replicates**	124	164	172	126	146
**Proteins identified in 3 replicates**	137	172	185	139	159

Among the detected proteins, 33% and 36%, which correspond to 205 proteins (normal) or 90 proteins (CKD), are identified by all methods (S6 Fig). These include many highly-abundant proteins such as albumin, vitamin D-binding protein, clusterin, zinc-alpha-2-glycoprotein, uromodulin and beta-2-microglobulin (S2 Table). This "core proteome" corresponds to 53% (+/-7%; normal) and 58% (+/-8%; CKD) of total identifications received per method. In fact, these common proteins correspond to approx. 95% of the total protein peak area in all analyzed samples. The percentage of identified proteins that are unique per analysis method is low, in the range of 10–15% (S2 Table).

To confirm efficiency of analysis, the applied LC-MS/MS protocol was compared to various alternative experimental conditions including: Top 20 versus Top 10 or Top 7 MS/MS analysis; injection of 1 versus 4 μg of protein. In all cases no substantial difference to the presented data could be observed. Importantly, the applied protocol provided average numbers of received MS/MS scans similar to numbers reported in published high resolution datasets [37, 38].

### Changes in protein sequence coverage after protein depletion

Peptide sequences per protein identified from the five technical replicates were combined and used for coverage calculations (S2 Table). The coverage from depleted samples was compared with the coverage from initial samples (log_2_ ratio depleted/initial urine) (S7–S10 Figs). For all samples from healthy controls, the depletion reduced sequence coverage of protein targets compared to the undepleted urine (from 5% reduction for IgG, up to 90% for serotransferrin in Seppro IgY14 kit). Similarly, in the case of CKD samples, sequence coverage slightly decreased for all depletion targets after application of the albumin and IgG depletion kits (ProteoPrep and SpinTrap) (S8 and S9 Figs). Decrease in the sequence coverage of three target proteins was not observed after fractionation through Seppro IgY14 (S7 Fig): albumin, alpha-1-acid glycoprotein 1 and immunoglobulin alpha. Similarly, sequence coverage did not decrease for alpha-1-antitrypsin after applying ProteoSpin kit (S10 Figs). Among the non-target proteins, no clear trend or impact on sequence coverage could be observed following application of depletion strategies (S7–S10 Figs).

To further investigate this issue, the number of PSMs in relation to sequence coverage was studied. A positive correlation between protein sequence coverage and PSMs could be observed in all cases: if the sequence coverage for a given protein was higher in the depleted sample compared to the unfractionated urine, so was the number of respective PSMs. Similarly, decrease in protein sequence coverage was associated with lower number of PSMs (data not shown). This correlation was in the range of 60%-70% for normal and 70%-80% for CKD samples.

### Changes in relative abundance after protein depletion

**Fig 4:**
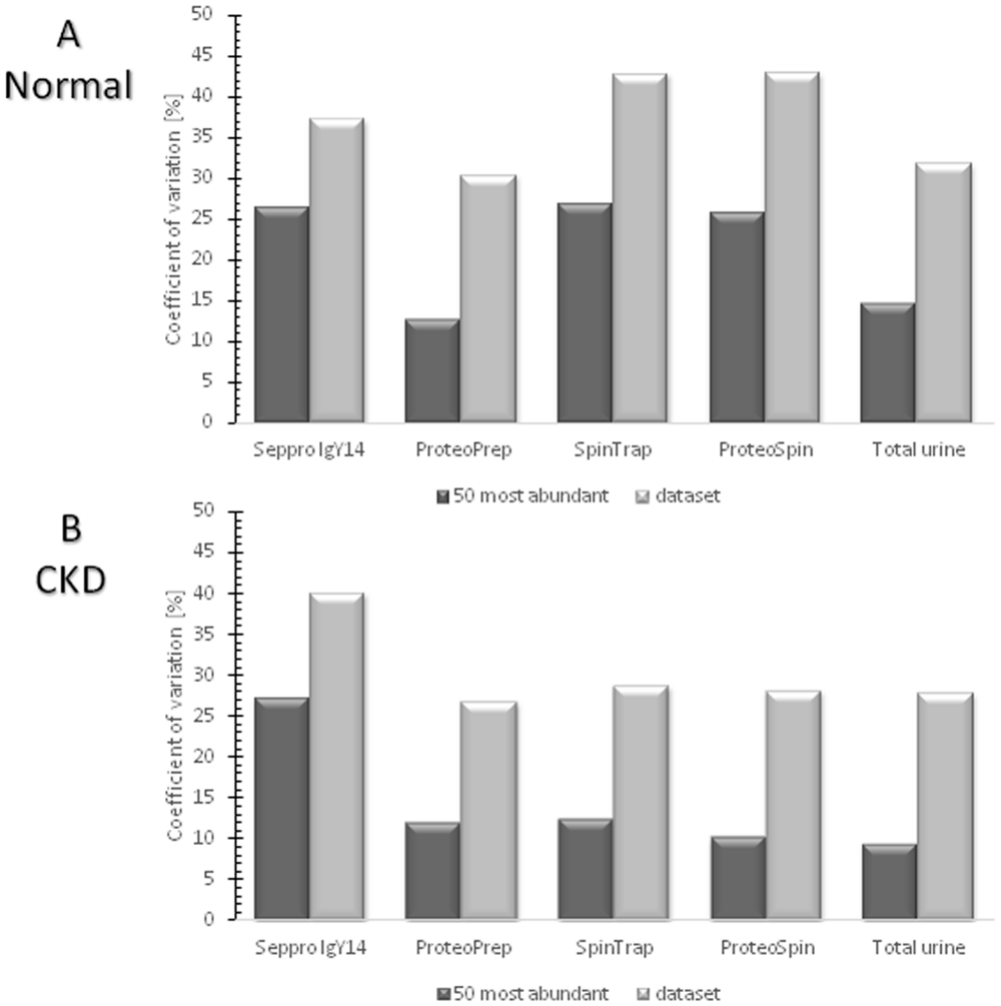
Coefficient of variation for 50 most abundant proteins and whole dataset for A) Normal, B) CKD urine. Normal samples appear having higher variability compared to the CKD samples, nevertheless this difference is not significant. Additionally and as expected, the variability increases when low-abundance proteins are included in the CV calculations.

To estimate the variability in protein abundance between technical replicates, the coefficient of variation for the 50 most abundant proteins from each sample and for the whole protein dataset was calculated (Fig 4). The list of 50 most abundant proteins per method tested is summarized in S3 Table. In the normal urine sample, higher variability was observed for Seppro IgY14, SpinTrap and ProteoSpin (CVs in the range of 26% for 50 most abundant and 40% for whole dataset). ProteoPrep and initial urine demonstrated variabilities in the range of 14% for the 50 most abundant, and 30% for the whole dataset. In the case of CKD samples, all of the analysis strategies demonstrated similar CVs (approx. 10% for 50 most abundant and 28% for the whole dataset), with the exception of Seppro IgY14, which showed a higher CV (27% for the 50 most abundant and 40% for the whole dataset). In all cases the variability increases (by approxiamately 16% for the 50 most abundant proteins) when low-abundance proteins are included in the CV calculations.

**Fig 5:**
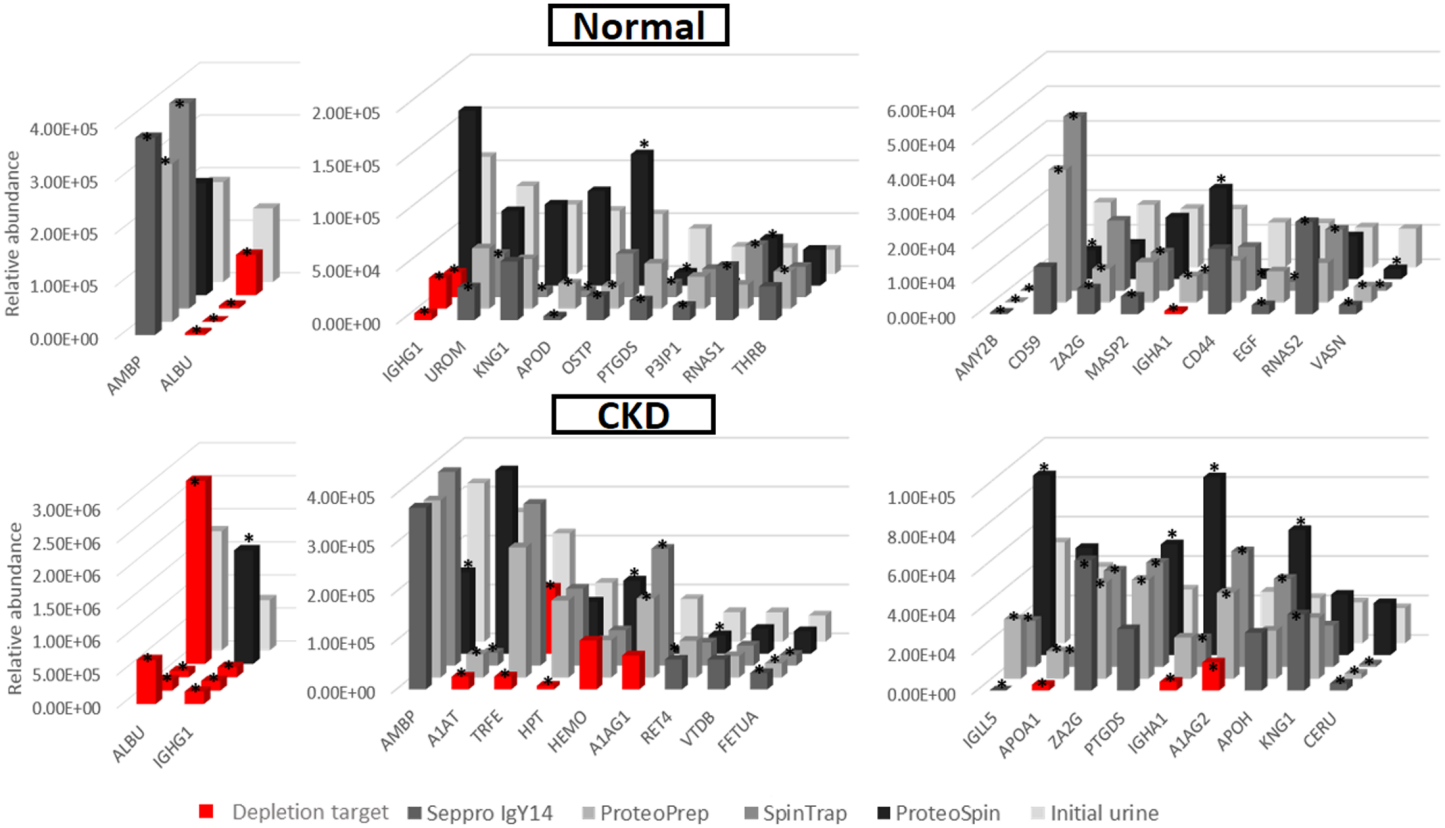
Relative abundance of 20 most abundant proteins derived from undepleted urine and comparison of their abundance with corresponding depleted fractions for urine from healthy controls and CKD patients. Efficient depletion of target proteins is observable for all methods, with the exception of albumin for ProteoSpin in CKD sample. * Denotes significant changes compared to initial urine. ABMP: protein AMBP, ALBU: albumin, IGHG1: Ig gamma-1 chain region, UROM: uromodulin, KNG1: kininogen 1, APOD: apolipoprotein D, OSTP: osteopontin, PTGDS: prostaglandin-H2 D-isomerase, P3IP1: phosphoinositide-3-kinase-interacting protein 1, RNAS1: ribonuclease pancreatic, THRB: prothrombin, AMY2B: alpha-amylase 2B, CD59: CD59 glycoprotein, ZA2G: zinc-alpha-2-glycoprotein, MASP2: mannan-binding lectin serine protease 2, IGHA1: Ig alpha-1 chain C region, CD44: CD44 antigen, EGF: pro-epidermal growth factor, RNAS2: non-secretory ribonuclease, VASN: vasorin, A1AT: alpha-1-antitrypsin, TRFE: serotransferrin, HPT: haptoglobin, HEMO: hemopexin, A1AG1: alpha-1-acid glycoprotein 1, RET4: retinol-binding protein 4, VTDB: vitamin D-binding protein, FETUA: alpha-2-HS-glycoprotein, IGLL5: immunoglobulin lambda-like polypeptide 5, APOA1: apolipoprotein A-I, A1AG2: alpha-1-acid glycoprotein 2, APOH: beta-2-glycoprotein 1, CERU: ceruloplasmin.

To evaluate the effect of depletion on relative abundance of proteins, a comparison of relative abundance of individual proteins between a depletion method and the undepleted urine was conducted. The enrichment or depletion of proteins was calculated based on the log_2_ ratio of signal intensity in the depleted against initial urine (S11–S14 Figs). Additionally, in Fig 5 the relative abundance of 20 most abundant proteins from undepleted urine (for normal and CKD) was compared to their abundance from corresponding depleted fractions. In the case of depletion targets, the application of immuno-based methods resulted in the reduction of their relative abundance. This observation is valid for urine from both normal and CKD patients. However, for the ion-exchange method (S14 Fig), the depletion was not efficient for serotransferrin in normal urine and for alpha-1-antitrypsin and albumin for CKD. When non-target proteins were compared, no clear trend in the abundance (increase or decrease) was observed. Collectively, similarly to protein sequence coverage, protein depletion had a variable impact on protein abundance, suggesting no added value of these strategies for the analysis of urine samples.

The depletion efficiency of the tested kits was also further estimated as follows: the relative abundance of albumin, as a target for all depletion kits, was compared before and after application of the fractionation strategies. As shown in Fig 5, significant depletion of Albumin was observed for normal samples: (approx. 98% decrease for all three immuno-based methods and 45% decrease for ion-exchange). For the urine from CKD patients, the most efficient depletion was observed for the albumin and IgG depletion kits: SpinTrap ProteoPrep and (95% and 91% decrease respectively), followed by the Seppro IgY14 (63% decrease). The depletion was inefficient in case of using ion-exchange ProteoSpin kit. Collectively, immuno-based methods outperformed the ion-exchange-based strategy in depleting albumin. Additionally, all three immuno-depletion kits depleted albumin with similar efficiency in the case of normal samples, whereas albumin and IgG depletion kits (ProteoPrep and SpinTrap) demonstrated higher depletion effectiveness compared to Seppro IgY14 for CKD. These results are in agreement with the SDS-PAGE analysis (Fig 2), where the highest albumin band intensity reduction was observed for ProteoPrep and SpinTrap, followed by Seppro IgY14 (see SDS-PAGE Analysis section). Of note, the relative abundance of Albumin based on MS data is noticeably higher in the initial CKD sample (approx. 65% of the total peak area) compared to normal (approx. 25% of the total peak area).

## Discussion

The main goal of the study was to evaluate the applicability of depletion of abundant proteins in urine samples from CKD patients and controls, at starting volumes regularly available from large clinical cohorts, using commercially available kits, originally designed for plasma. Based on the gel profiles from SDS-PAGE and the number of identified peptides from LC-MS/MS, each depletion strategy is reproducible, and in the case of normal samples, albumin as a target protein is efficiently depleted. For CKD samples, immunodepletion kits efficiently depleted albumin and the highest efficiency was observed for albumin and IgG depletion kits (ProteoPrep and SpinTrap) followed by Seppro IgY14 (Fig 2 and Fig 5).

The reduced efficiency of Seppro IgY14 may be attributed to potential column overloading-even though this was not expected to be the case based on the manufacturer’s instructions: Seppro IgY14 is designed to work with plasma, where the concentration of highly-abundant proteins is substantial. Additionally, the loaded protein amount in this study (437 μg) was not even half of the column binding capacity (1 mg max. column binding capacity). The reason(s) of the lower efficiency of Seppro IgY14 in depleting albumin in CKD urine is still unknown. The ion-exchange-based ProteoSpin kit was found to be the least efficient in eliminating target proteins from both normal and CKD urine. This was expected due to the highly-specific nature of immuno-based mechanism employed in the other kits [15].

Regardless whether a depletion method was applied or not, the number of protein identifications from LC-MS/MS analysis were comparable. In all cases, in the urine from CKD patients fewer proteins were identified in comparison to urine from healthy controls, even though the number of PSMs, MS/MS scans and TICs were similar per method. This may indicate that, even upon depletion, the potential masking effect from highly abundant proteins still exists. After depletion of the target highly-abundant proteins, other non-targeted high and medium-abundance molecules (e.g. protein AMBP, vitamin D-binding proteins, zinc-alpha-2-glycoprotein, uromodulin) likely maintain the masking effect. Alternatively, a large number of proteins may be below the limit of detection (estimated at low femtomole range for the applied mass spectrometer) and therefore, any positive impact of depletion on proteome coverage cannot be observed. Collectively, comparable numbers of received identifications between different strategies, as well as the presence of unique proteins in both fractionated and initial urine indicate no benefit of depletion for biomarker identification purposes.

Our results are not in agreement with Kushnir et al. [30] findings, where the employment of a multiple affinity removal (MARS) column allowed increasing the number of identifications in urine from 60 to 142 in CKD patients. Still, in our presented study the number of protein identifications is higher in comparison, possibly a result of a less sensitive instrument used by the authors (Q-TOF equipped with a ChipCube). The immuno-based depletion strategies were also evaluated in 2D gel proteomics experiments [24, 27, 39, 40]. In these cases the number of unique identifications did not change significantly following depletion.

In order to evaluate the validity of the obtained protein identifications from urine from healthy controls, 100 most abundant (as the most reliable) proteins from each analysis method (i.e. undepleted and fractionated samples), were compared with the identifications from three manuscripts reporting on the analysis of urine proteome from healthy individuals [41–43]. In each case, approx. 90 out of the 100 most abundant proteins identified in the present study were also reported in these manuscripts. When expanding the comparison from the 100 most abundant to the whole dataset an overlap of approx. 60%, for the normal samples was observed, similar to the overlap of protein identifications between the three different studies. These similarities between different datasets representing normal urine support the validity of our data. To estimate the validity of obtained identifications from CKD samples, proteins from all CKD datasets were compared with molecules associated with renal diseases reported in the literature [44–46]. Due to the too small sample size tested to evaluate differential expression of these molecules, our focus was set only on their presence. Several of these disease-associated proteins were identified in all datasets (i.e. albumin, neutrophil gelatinase-associated lipocalin, cystatin C, osteopontin, clusterin, beta-2-microglobulin). A few were unique for applied strategies: metalloproteinase inhibitor 1 was present in three kits (Seppro IgY14, SpinTrap and ProteoSpin), fatty acid-binding protein was unique for unfractionated sample and connective tissue growth factor for SpinTrap kit.

Based on the available literature data and in line to our observations, a number of non-targeted proteins are also depleted to some degree [23–25], negatively affecting the analysis. This effect may be related to the fact that targeted proteins may form stable complexes with non-targeted proteins resulting in their co-depletion. This co-depletion mechanism was observed in a number of studies (i.e. [23–25]). For example, Granger et al. [23] demonstrated that depletion of albumin removed also low-abundance proteins including cytokines from plasma samples. Similarly, Stempfer et al. [25], spiked 6 recombinant cytokines in serum samples and showed that application of depletion methods reduced the cytokine levels.

The application of depletion strategies did not improve the proteome or sequence coverage. Given that the overall data quality and quantity (as reflected by the number of MS/MS scans—Table 2) were not significantly affected following fractionation, the fact that no clear increase in proteome and sequence coverage could be observed may be attributed to the following factors: proteome complexity rendering effects of depletion per protein are unpredictable, lack of sufficient depletion to generate an observable impact on coverage as well as peptides (even if enriched) still remaining below the limit of detection (i.e. undersampling at an individual protein level).

Comparison of changes in protein abundance for overlapping identifications prior to and after depletion was also performed by Tu et al. [26] for plasma samples using MARS columns. In contrast to the present study, where no clear advantage of protein depletion was observed, the authors found that most of the non-targeted proteins were enriched after depletion. This discrepancy may be related to: i) depletion was evaluated in plasma samples, not urine, and ii) significantly higher starting protein content was used.

Target proteins were less efficiently depleted in the urine from CKD patients compared to normal, regardless of the depletion strategy applied, even though the protein content loaded onto the depletion column was always (according to the manufacturers protocols) below their loading capacity. However, it may be that the actual loading capacity is lower than claimed.

In conclusion, the depletion of abundant proteins does not present an added value for the study of the urine proteome, at least when starting with small urine volumes (less than 1 mL), regularly available in large clinical studies. No significant improvement in the number of identifications, protein sequence coverage or relative abundance in comparison to the undepleted samples were detected using different methods in the current study. Moreover, the depletion introduced additional variability. Depletion of targeted proteins was substantially more efficient in normal than for CKD samples, suggesting that additional disease-related factors may impair the depletion efficiency. Therefore, for the urinary proteomics studies especially in the context of CKD, analysis of total rather than depleted urine appears preferable.

## Supporting Information

S1 Table Peptide lists for all analysis methods. (XLSX)

S2 Table Lists of common and unique identifications for depleted samples and initial urine for all strategies. (XLSX)

S3 Table 50 most abundant proteins for each depletion strategy and unfractionated sample for normal and CKD urine. X denotes that the protein was found as one of the 50 most abundant in the respective analysis method.
(XLSX)

S1 Fig SDS-PAGE gel profiles for Seppro IgY14 depletion kit for normal samples: Depleted fractions and albumin as a target protein are marked. M—molecular size marker. I—initial urine. F—Flow-through fraction. W—Wash. E—Elution. 1–4 –consecutive numbers of flow-through/wash/elution within one replicate.
(TIF)

S2 Fig SDS-PAGE gel profiles for Seppro IgY14 depletion kit for CKD samples: Depleted fractions and albumin as a target protein are marked. M—molecular size marker. I—initial urine. F—Flow-through fraction. W—Wash. E—Elution. 1–4 –consecutive numbers of flow-through/wash/elution within one replicate.
(TIF)

S3 Fig SDS-PAGE gel profiles for ProtePrep depletion kit. Depleted fractions and albumin as a target protein are marked. M—molecular size marker. I—initial urine. F—Flow-through fraction. E—Elution. I-V—number of technical replicate.
(TIF)

S4 Fig SDS-PAGE gel profiles for SpinTrap depletion kit. Depleted fractions and albumin as a target protein are marked. M—molecular size marker. I—initial urine. F—Flow-through fraction. E—Elution. I-V—number of technical replicate.
(TIF)

S5 Fig SDS-PAGE gel profiles for ProteoSpin depletion kit. Depleted fractions and albumin as a target protein are marked. M—molecular size marker. I—initial urine. F—Flow-through fraction. E—Elution. I-V—number of technical replicate.
(TIF)

S6 Fig Venn Diagram [47]: unique identifications for urine from A) normal B) CKD patients. In total 612 and 251 unique proteins, in at least three out of five replicates, were identified in normal and CKD samples respectively. Approximately 33% of the identifications are shared between non-depleted and depleted urine in normal or CKD sample.
(TIF)

S7 Fig Changes in protein sequence coverage for overlapping identifications between Seppro IgY14 depleted sample and initial urine. X axis represents the protein sequence coverage of the initial urine. The changes after applying the depletion strategy are presented on Y-axis (with log_2_ scale) as a ratio of depleted versus non-depleted sample. Proteins, with increased sequence coverage are presented above the ratio of 0 on the Y-scale and with decreased below the ratio of 0. Proteins with a ratio of 0 show the same coverage in the initial and depleted sample. Sequence coverage for immunoglobulins is presented as an average coverage for all proteins combined in the group. Protein targets for depletion kit are marked as red dots (see Table 1). Protein targets for which the protein sequence coverage increased after depletion are marked by an arrow.
(TIF)

S8 Fig Changes in protein sequence coverage for overlapping identifications between ProteoPrep depleted sample and initial urine. X axis represents the protein sequence coverage of the initial urine. The changes after applying the depletion strategy are presented on Y-axis (with log_2_ scale) as a ratio of depleted versus non-depleted sample. Proteins, with increased sequence coverage are presented above the ratio of 0 on the Y-scale and with decreased below the ratio of 0. Proteins with a ratio of 0 show the same coverage in the initial and depleted sample. Sequence coverage for immunoglobulins is presented as an average coverage for all proteins combined in the group. Protein targets for depletion kit are marked as red dots (see Table 1).
(TIF)

S9 Fig Changes in protein sequence coverage for overlapping identifications between SpinTrap depleted sample and initial urine. X axis represents the protein sequence coverage of the initial urine. The changes after applying the depletion strategy are presented on Y-axis (with log_2_ scale) as a ratio of depleted versus non-depleted sample. Proteins, with increased sequence coverage are presented above the ratio of 0 on the Y-scale and with decreased below the ratio of 0. Proteins with a ratio of 0 show the same coverage in the initial and depleted sample. Sequence coverage for immunoglobulins is presented as an average coverage for all proteins combined in the group. Protein targets for depletion kit are marked as red dots (see Table 1).
(TIF)

S10 Fig Changes in protein sequence coverage for overlapping identifications between ProteoSpin depleted sample and initial urine. X axis represents the protein sequence coverage of the initial urine. The changes after applying the depletion strategy are presented on Y-axis (with log_2_ scale) as a ratio of depleted versus non-depleted sample. Proteins, with increased sequence coverage are presented above the ratio of 0 on the Y-scale and with decreased below the ratio of 0. Proteins with a ratio of 0 show the same coverage in the initial and depleted sample. Sequence coverage for immunoglobulins is presented as an average coverage for all proteins combined in the group. Protein targets for depletion kit are marked as red dots (see Table 1). Protein targets for which the protein sequence coverage increased after depletion are marked by an arrow.
(TIF)

S11 Fig Changes in protein abundance for overlapping identifications between Seppro IgY14 depleted sample and initial urine. The scatterplots present the protein relative abundance changes after protein depletion in comparison to the initial sample. X axis represents the normalized protein abundance for initial urine in logarithmic scale (log_2_). Proteins on the Y axis (log_2_ scale) above a ratio of 0 are enriched in comparison to initial urine, while those below the ratio of 0 are depleted. Proteins with a ratio 0 show the same relative abundance in the initial and depleted sample. Protein abundance for immunoglobulins is presented as a sum of the abundance for all combined proteins in the group. Protein targets for depletion kit are marked as red dots (see Table 1).
(TIF)

S12 Fig Changes in protein abundance for overlapping identifications between ProteoPrep depleted sample and initial urine. The scatterplots present the protein relative abundance changes after protein depletion in comparison to the initial sample. X axis represents the normalized protein abundance for initial urine in logarithmic scale (log_2_). Proteins on the Y axis (log_2_ scale) above a ratio of 0 are enriched in comparison to initial urine, while those below the ratio of 0 are depleted. Proteins with a ratio 0 show the same relative abundance in the initial and depleted sample. Protein abundance for immunoglobulins is presented as a sum of the abundance for all combined proteins in the group. Protein targets for depletion kit are marked as red dots (see Table 1).
(TIF)

S13 Fig Changes in protein abundance for overlapping identifications between SpinTrap depleted sample and initial urine. The scatterplots present the protein relative abundance changes after protein depletion in comparison to the initial sample. X axis represents the normalized protein abundance for initial urine in logarithmic scale (log_2_). Proteins on the Y axis (log_2_ scale) above a ratio of 0 are enriched in comparison to initial urine, while those below the ratio of 0 are depleted. Proteins with a ratio 0 show the same relative abundance in the initial and depleted sample. Protein abundance for immunoglobulins is presented as a sum of the abundance for all combined proteins in the group. Protein targets for depletion kit are marked as red dots (see Table 1).
(TIF)

S14 Fig Changes in protein abundance for overlapping identifications between ProteoSpin depleted sample and initial urine. The scatterplots present the protein relative abundance changes after protein depletion in comparison to the initial sample. X axis represents the normalized protein abundance for initial urine in logarithmic scale (log_2_). Proteins on the Y axis (log_2_ scale) above a ratio of 0 are enriched in comparison to initial urine, while those below the ratio of 0 are depleted. Proteins with a ratio 0 show the same relative abundance in the initial and depleted sample. Protein abundance for immunoglobulins is presented as a sum of the abundance for all combined proteins in the group. Protein targets for depletion kit are marked as red dots (see Table 1). Protein targets for which the relative abundance increased after depletion are marked by an arrow.
(TIF)
