# Supplementary figures and images for: Comparison of Depletion Strategies for the Enrichment of Low-Abundance Proteins in Urine
Source: PLoS One. 2015 Jul 24;10(7):e0133773. doi: 10.1371/journal.pone.0133773 (PMC4514849; doi:10.1371/journal.pone.0133773)

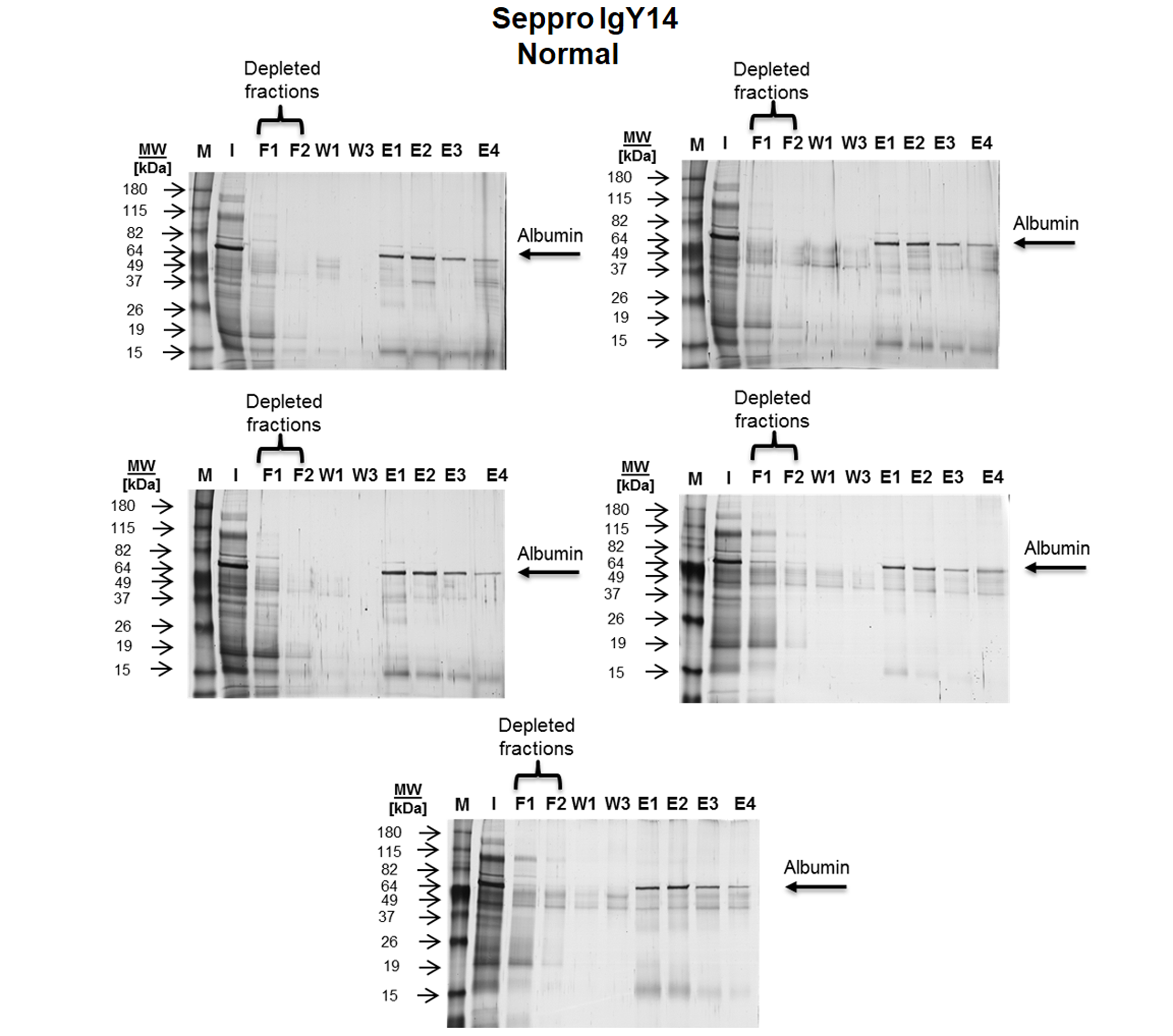

Supplement: S1 Fig — M—molecular size marker. I—initial urine. F—Flow-through fraction. W—Wash. E—Elution. 1–4 –consecutive numbers of flow-through/wash/elution within one replicate. (TIF) [file pone.0133773.s004.TIF]

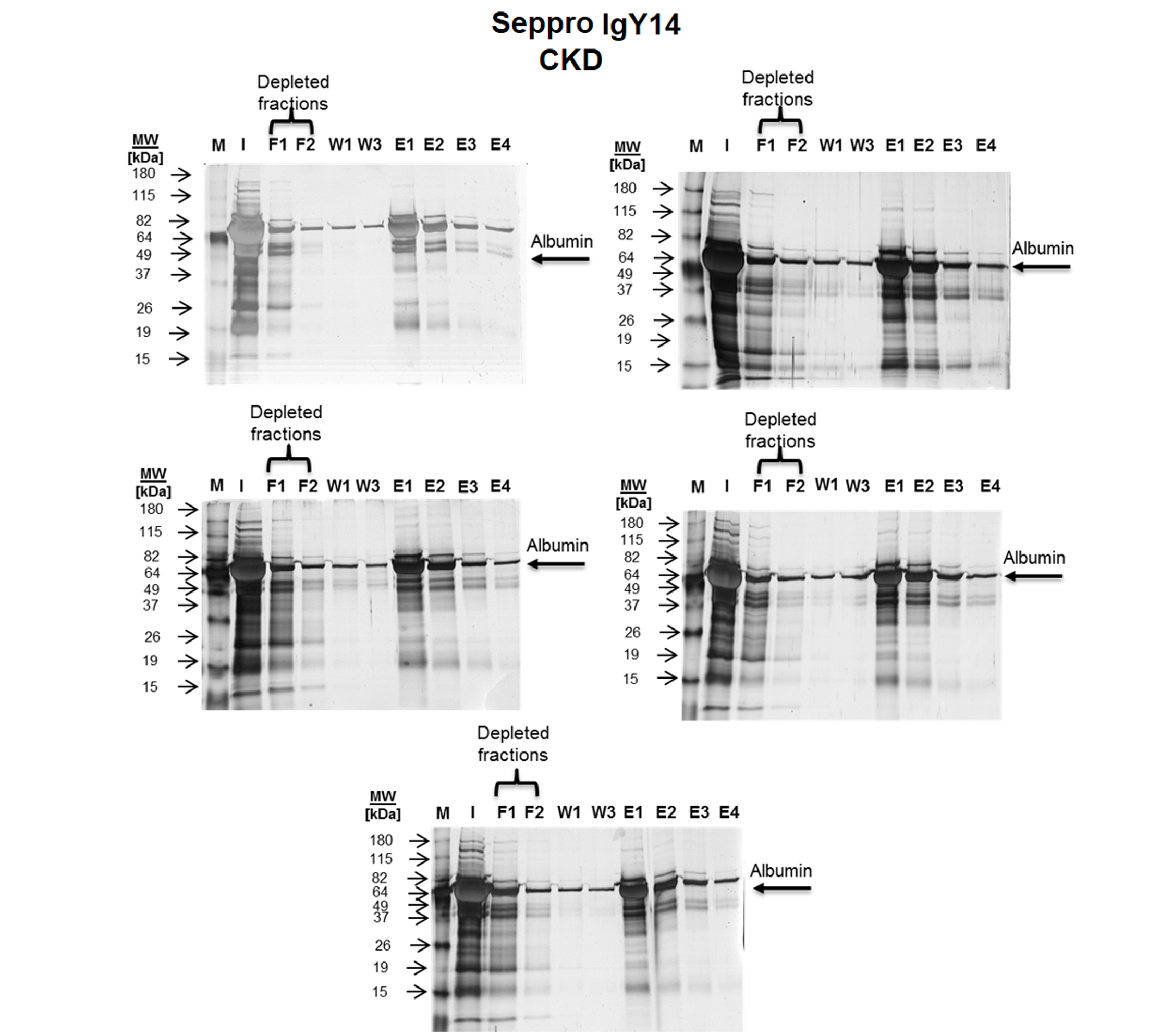

Supplement: S2 Fig — M—molecular size marker. I—initial urine. F—Flow-through fraction. W—Wash. E—Elution. 1–4 –consecutive numbers of flow-through/wash/elution within one replicate. (TIF) [file pone.0133773.s005.TIF]

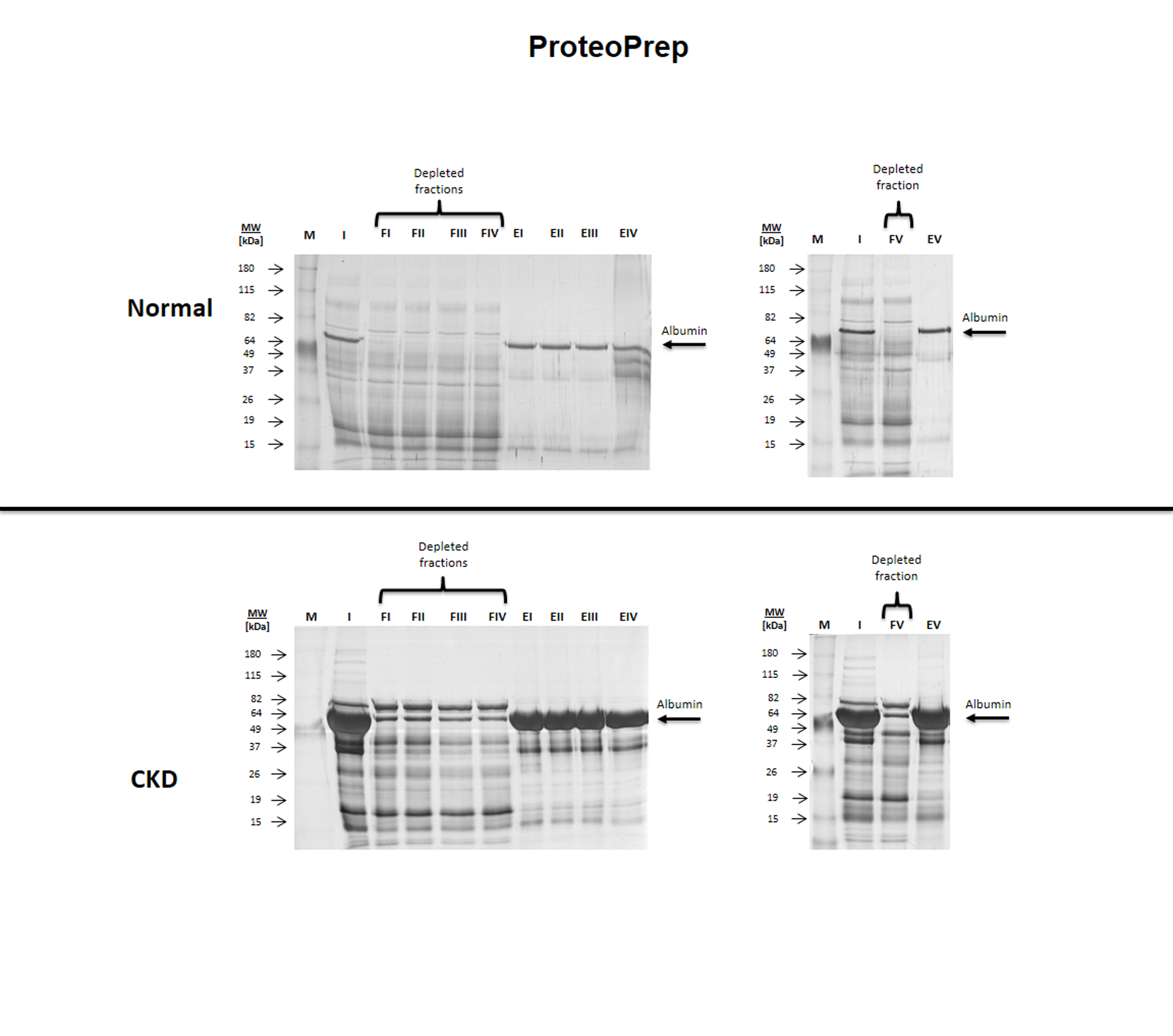

Supplement: S3 Fig — Depleted fractions and albumin as a target protein are marked. M—molecular size marker. I—initial urine. F—Flow-through fraction. E—Elution. I-V—number of technical replicate. (TIF) [file pone.0133773.s006.TIF]

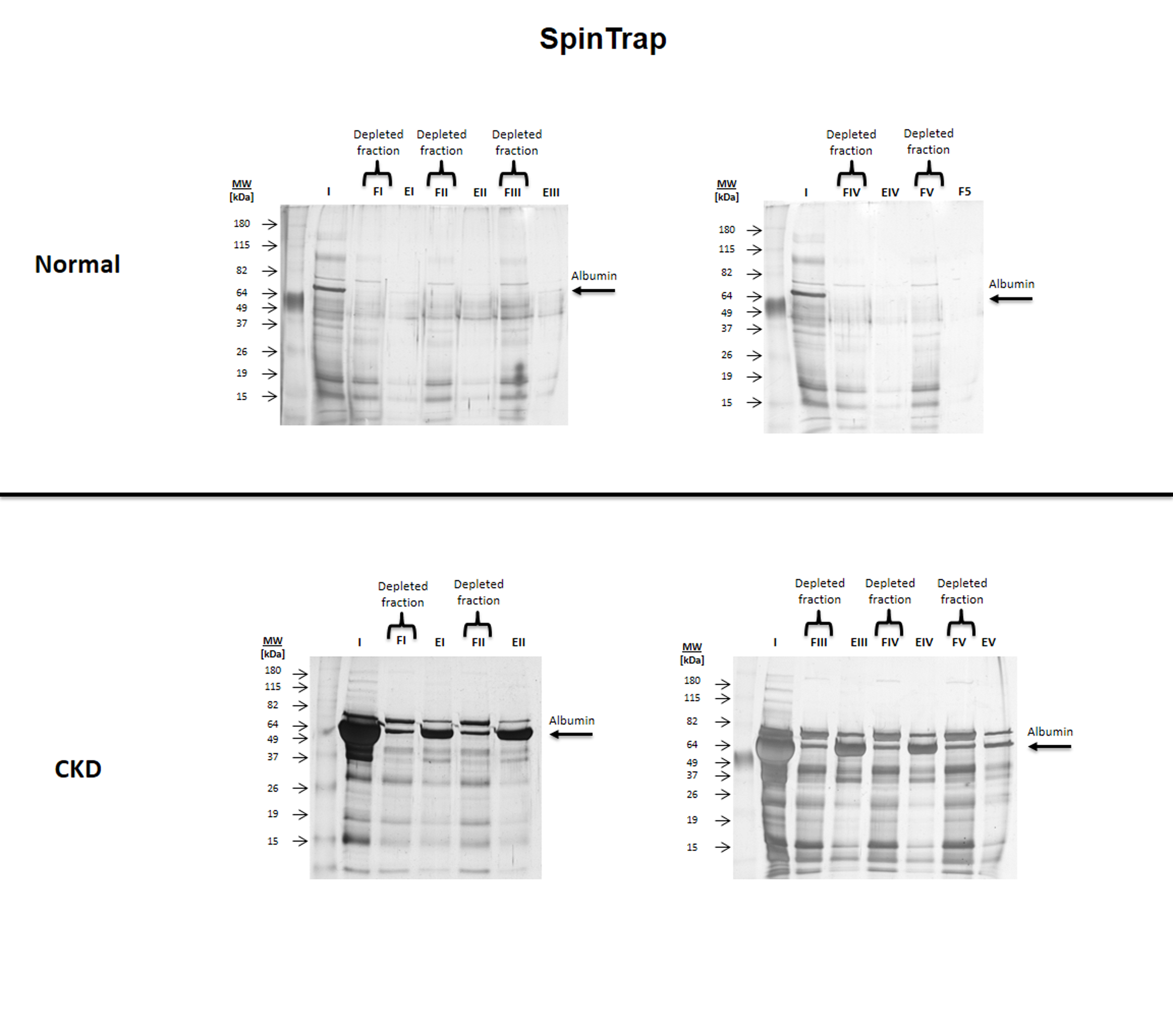

Supplement: S4 Fig — Depleted fractions and albumin as a target protein are marked. M—molecular size marker. I—initial urine. F—Flow-through fraction. E—Elution. I-V—number of technical replicate. (TIF) [file pone.0133773.s007.TIF]

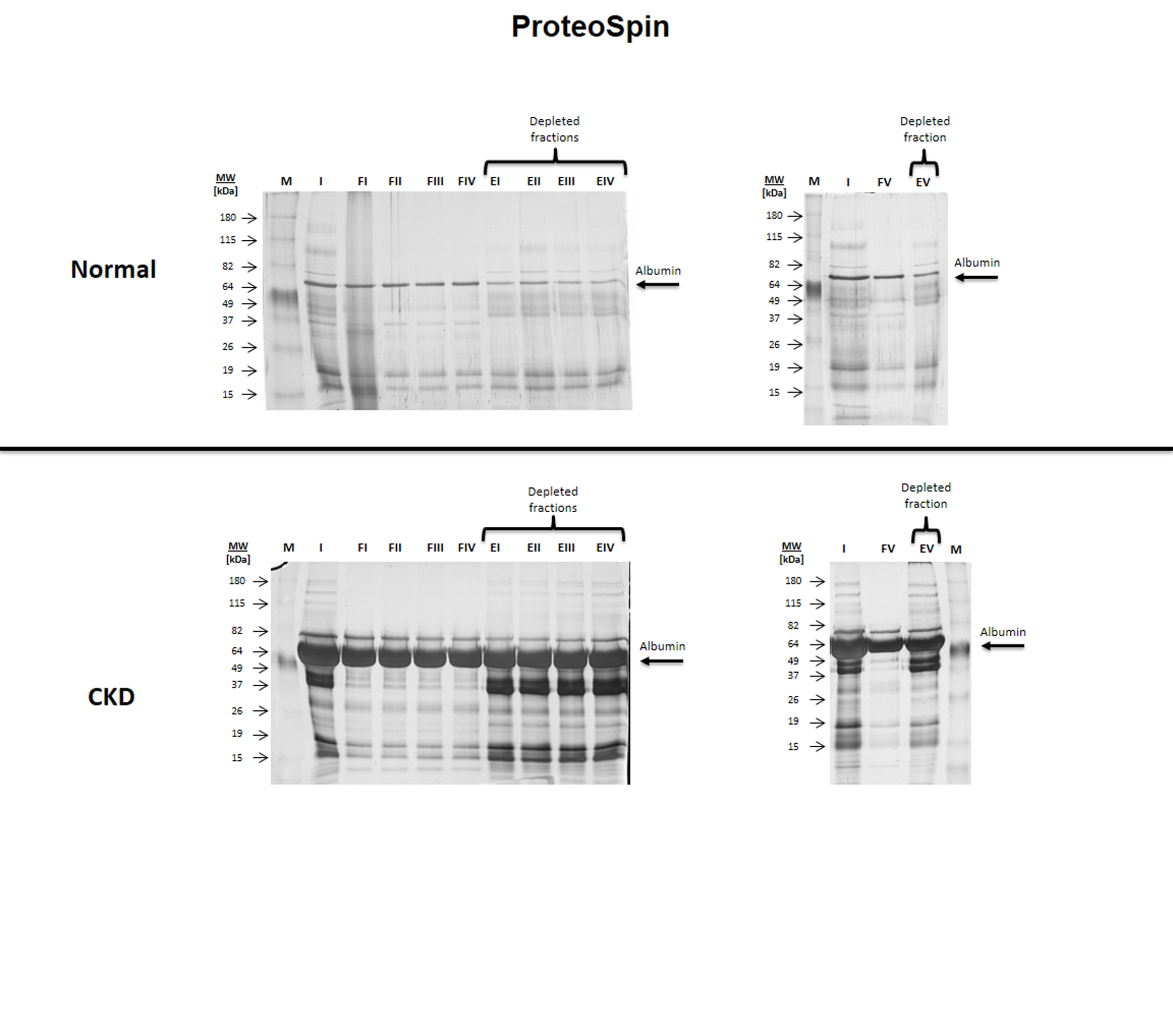

Supplement: S5 Fig — Depleted fractions and albumin as a target protein are marked. M—molecular size marker. I—initial urine. F—Flow-through fraction. E—Elution. I-V—number of technical replicate. (TIF) [file pone.0133773.s008.TIF]

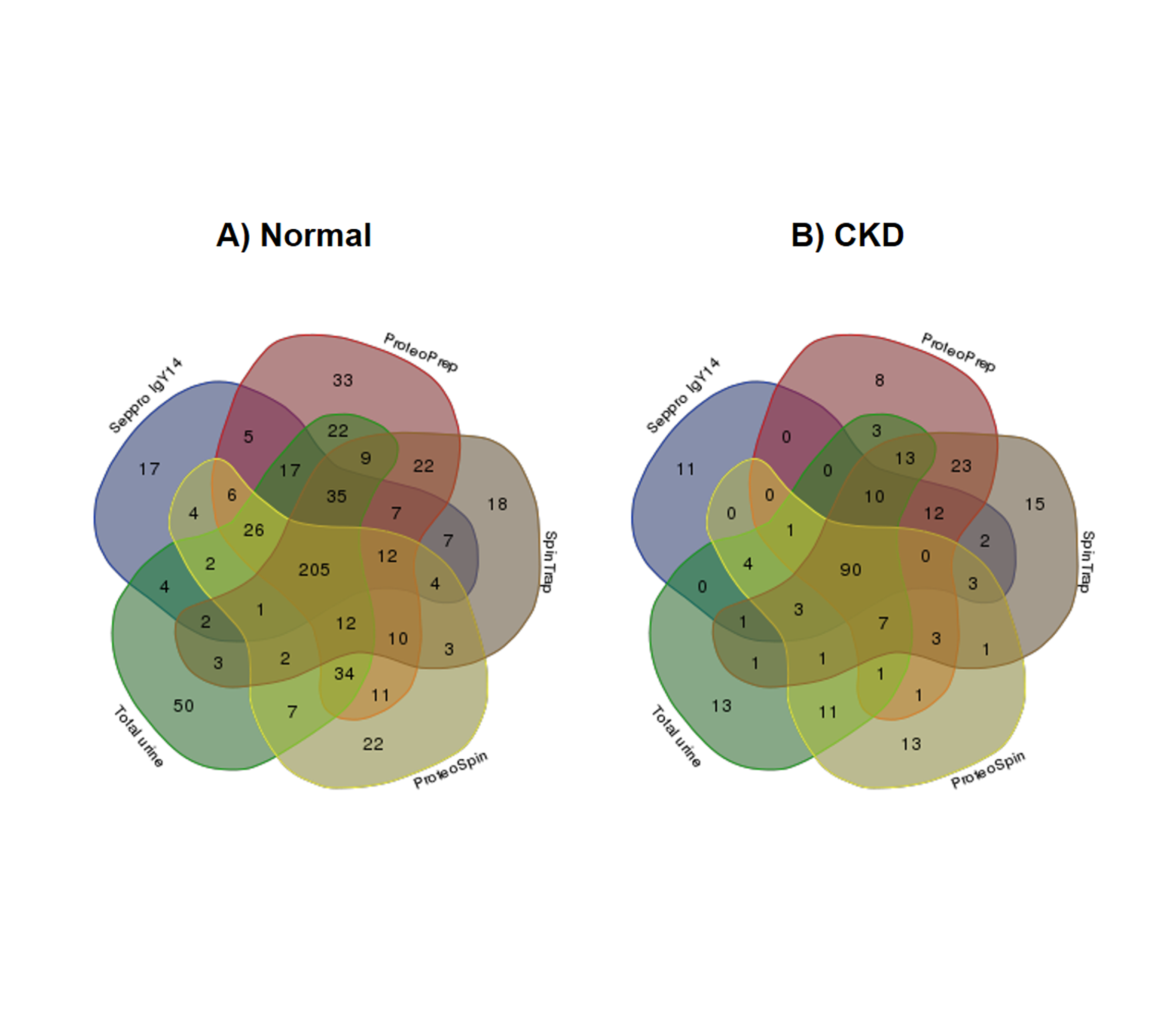

Supplement: S6 Fig — In total 612 and 251 unique proteins, in at least three out of five replicates, were identified in normal and CKD samples respectively. Approximately 33% of the identifications are shared between non-depleted and depleted urine in normal or CKD sample. (TIF) [file pone.0133773.s009.TIF]

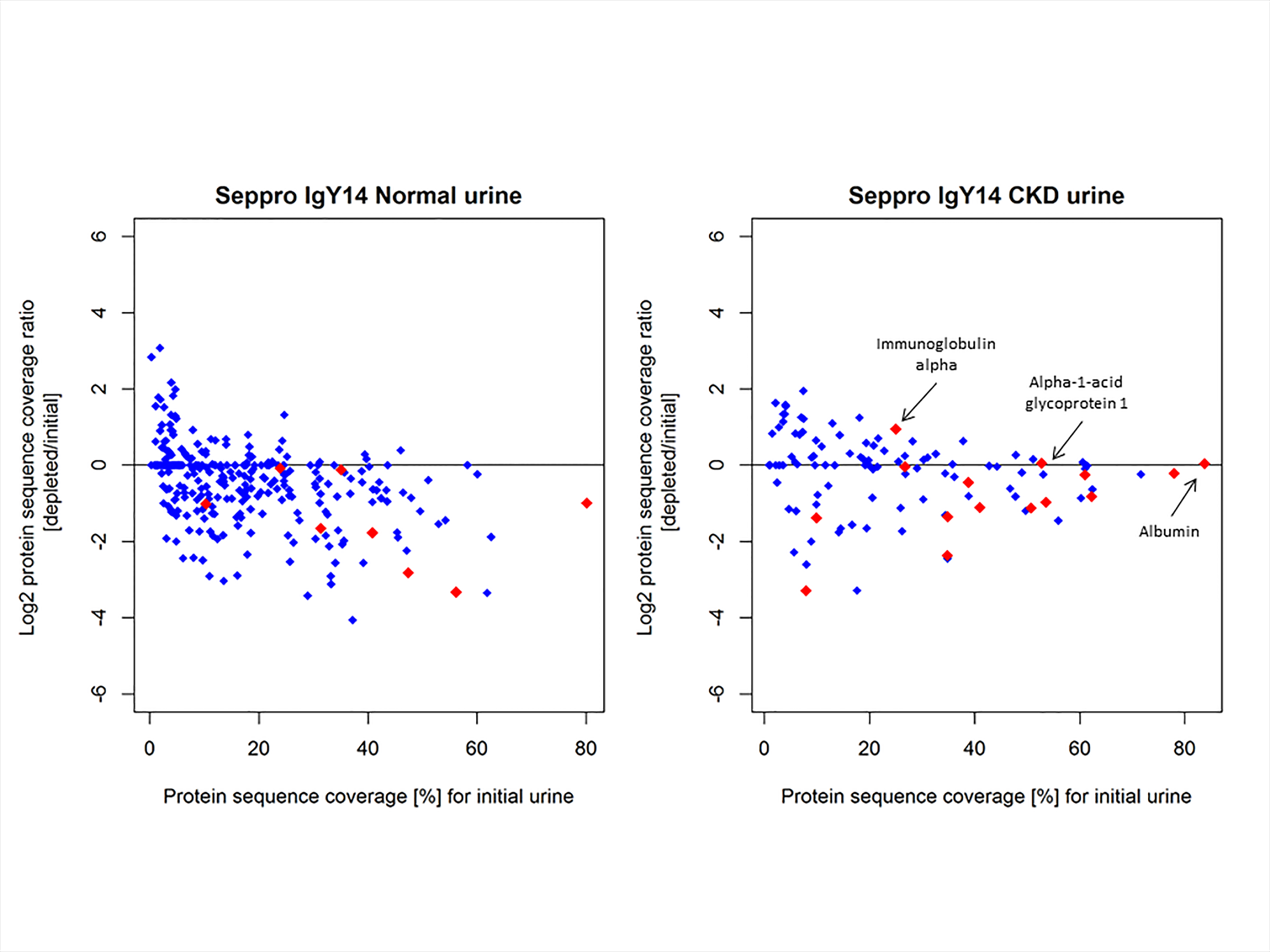

Supplement: S7 Fig — X axis represents the protein sequence coverage of the initial urine. The changes after applying the depletion strategy are presented on Y-axis (with log2 scale) as a ratio of depleted versus non-depleted sample. Proteins, with increased sequence coverage are presented above the ratio of 0 on the Y-scale and with decreased below the ratio of 0. Proteins with a ratio of 0 show the same coverage in the initial and depleted sample. Sequence coverage for immunoglobulins is presented as an average coverage for all proteins combined in the group. Protein targets for depletion kit are marked as red dots (see Table 1). Protein targets for which the protein sequence coverage increased after depletion are marked by an arrow. (TIF) [file pone.0133773.s010.TIF]

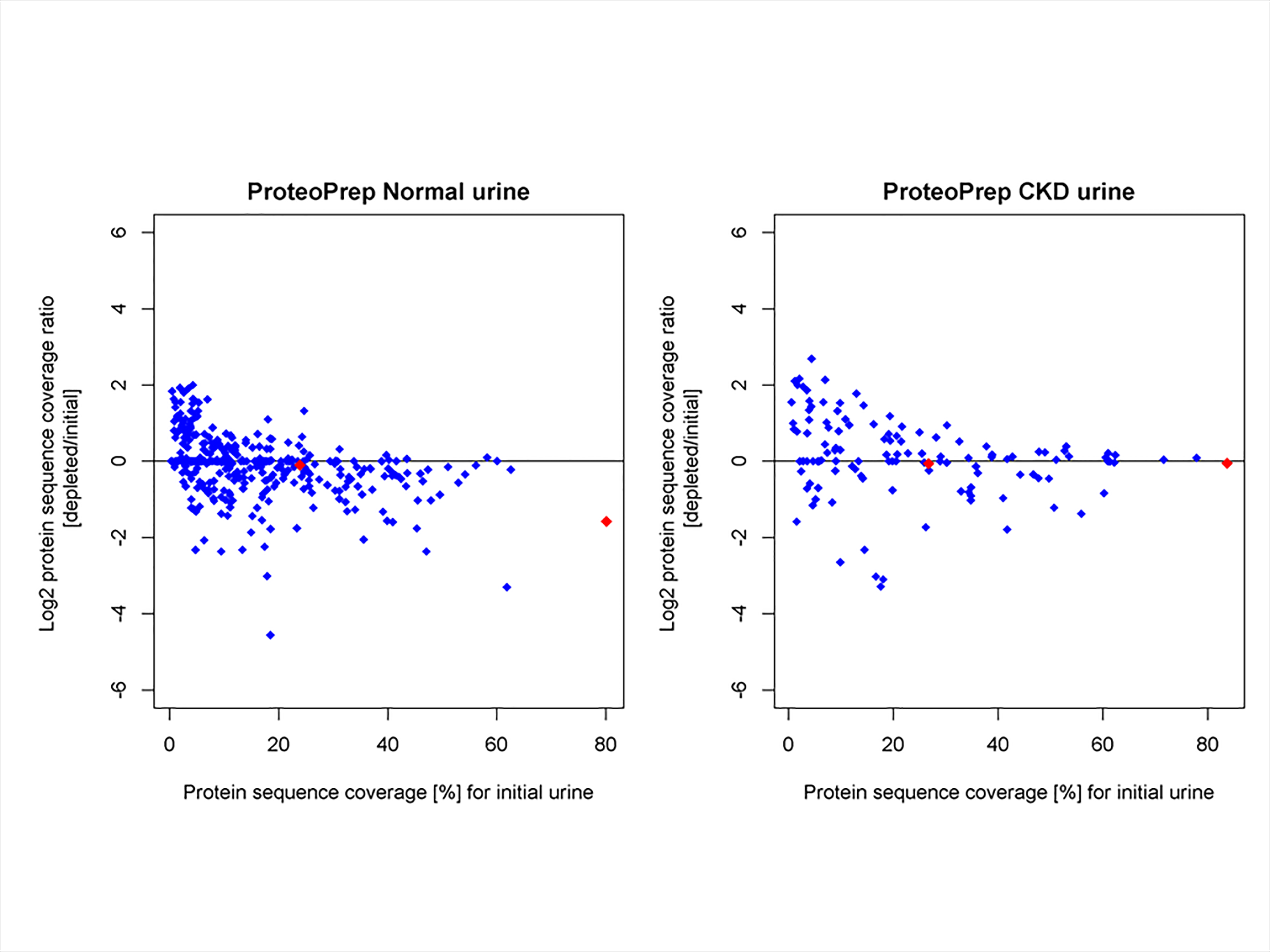

Supplement: S8 Fig — X axis represents the protein sequence coverage of the initial urine. The changes after applying the depletion strategy are presented on Y-axis (with log2 scale) as a ratio of depleted versus non-depleted sample. Proteins, with increased sequence coverage are presented above the ratio of 0 on the Y-scale and with decreased below the ratio of 0. Proteins with a ratio of 0 show the same coverage in the initial and depleted sample. Sequence coverage for immunoglobulins is presented as an average coverage for all proteins combined in the group. Protein targets for depletion kit are marked as red dots (see Table 1). (TIF) [file pone.0133773.s011.TIF]

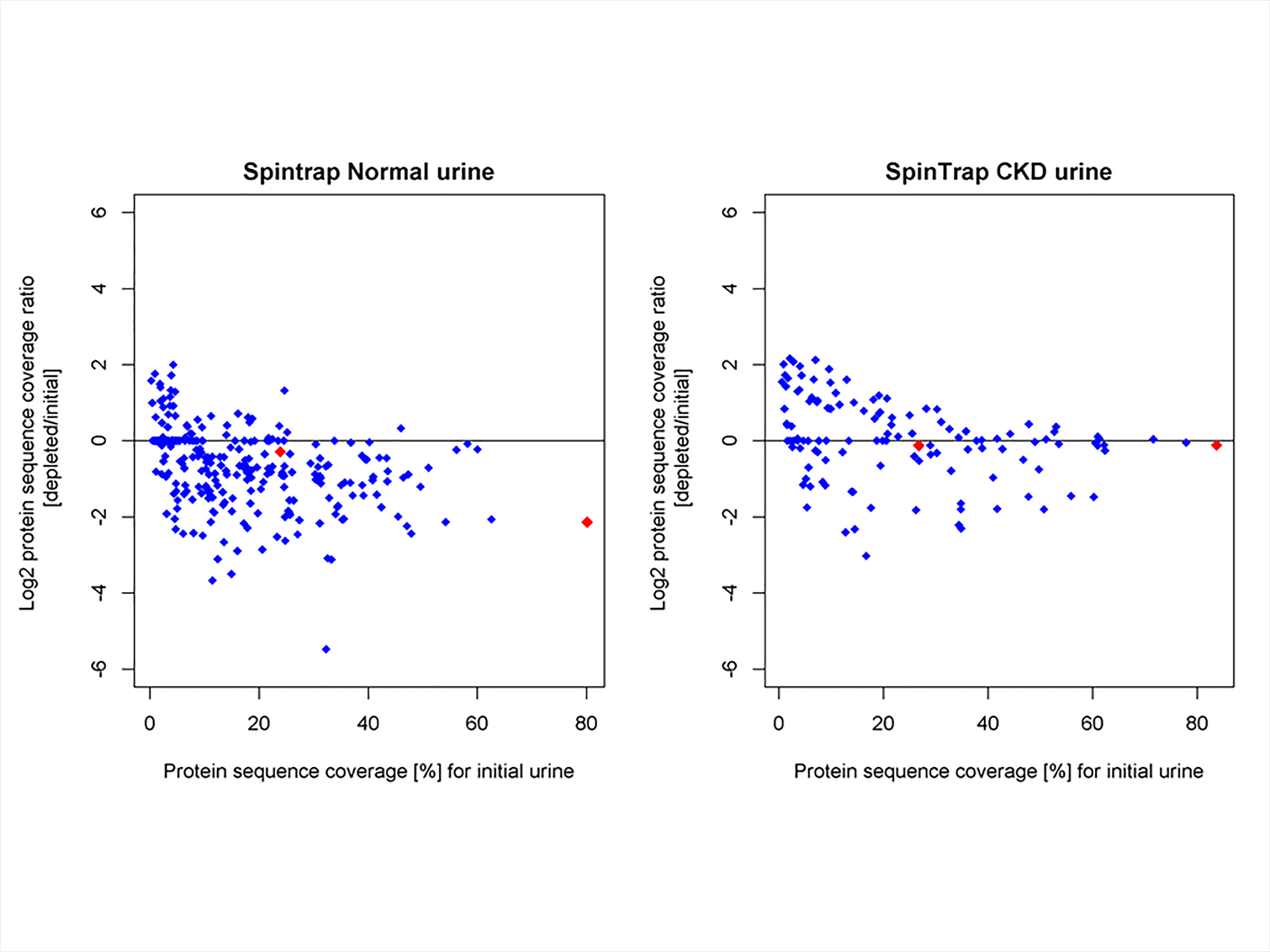

Supplement: S9 Fig — X axis represents the protein sequence coverage of the initial urine. The changes after applying the depletion strategy are presented on Y-axis (with log2 scale) as a ratio of depleted versus non-depleted sample. Proteins, with increased sequence coverage are presented above the ratio of 0 on the Y-scale and with decreased below the ratio of 0. Proteins with a ratio of 0 show the same coverage in the initial and depleted sample. Sequence coverage for immunoglobulins is presented as an average coverage for all proteins combined in the group. Protein targets for depletion kit are marked as red dots (see Table 1). (TIF) [file pone.0133773.s012.TIF]

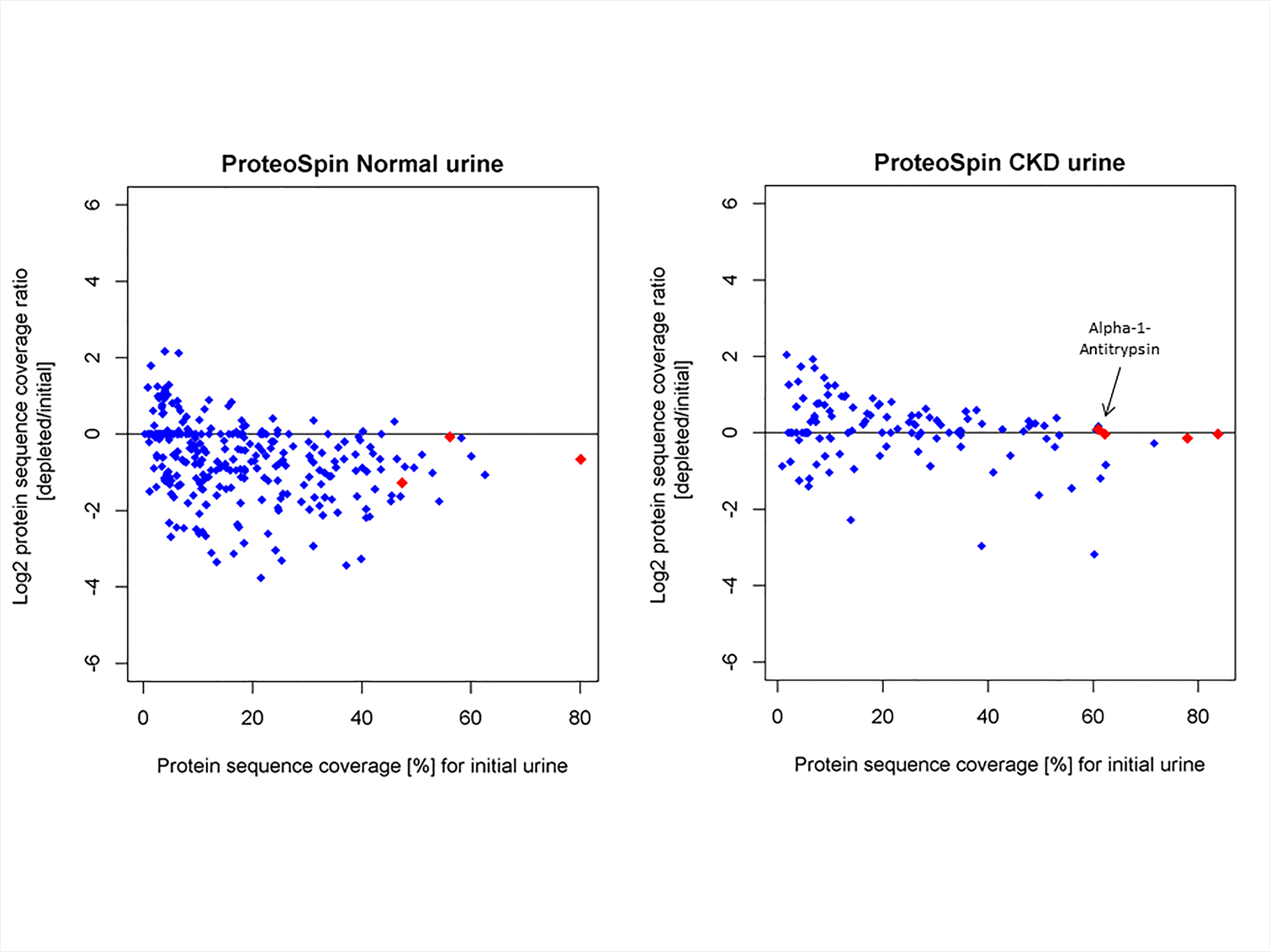

Supplement: S10 Fig — X axis represents the protein sequence coverage of the initial urine. The changes after applying the depletion strategy are presented on Y-axis (with log2 scale) as a ratio of depleted versus non-depleted sample. Proteins, with increased sequence coverage are presented above the ratio of 0 on the Y-scale and with decreased below the ratio of 0. Proteins with a ratio of 0 show the same coverage in the initial and depleted sample. Sequence coverage for immunoglobulins is presented as an average coverage for all proteins combined in the group. Protein targets for depletion kit are marked as red dots (see Table 1). Protein targets for which the protein sequence coverage increased after depletion are marked by an arrow. (TIF) [file pone.0133773.s013.TIF]

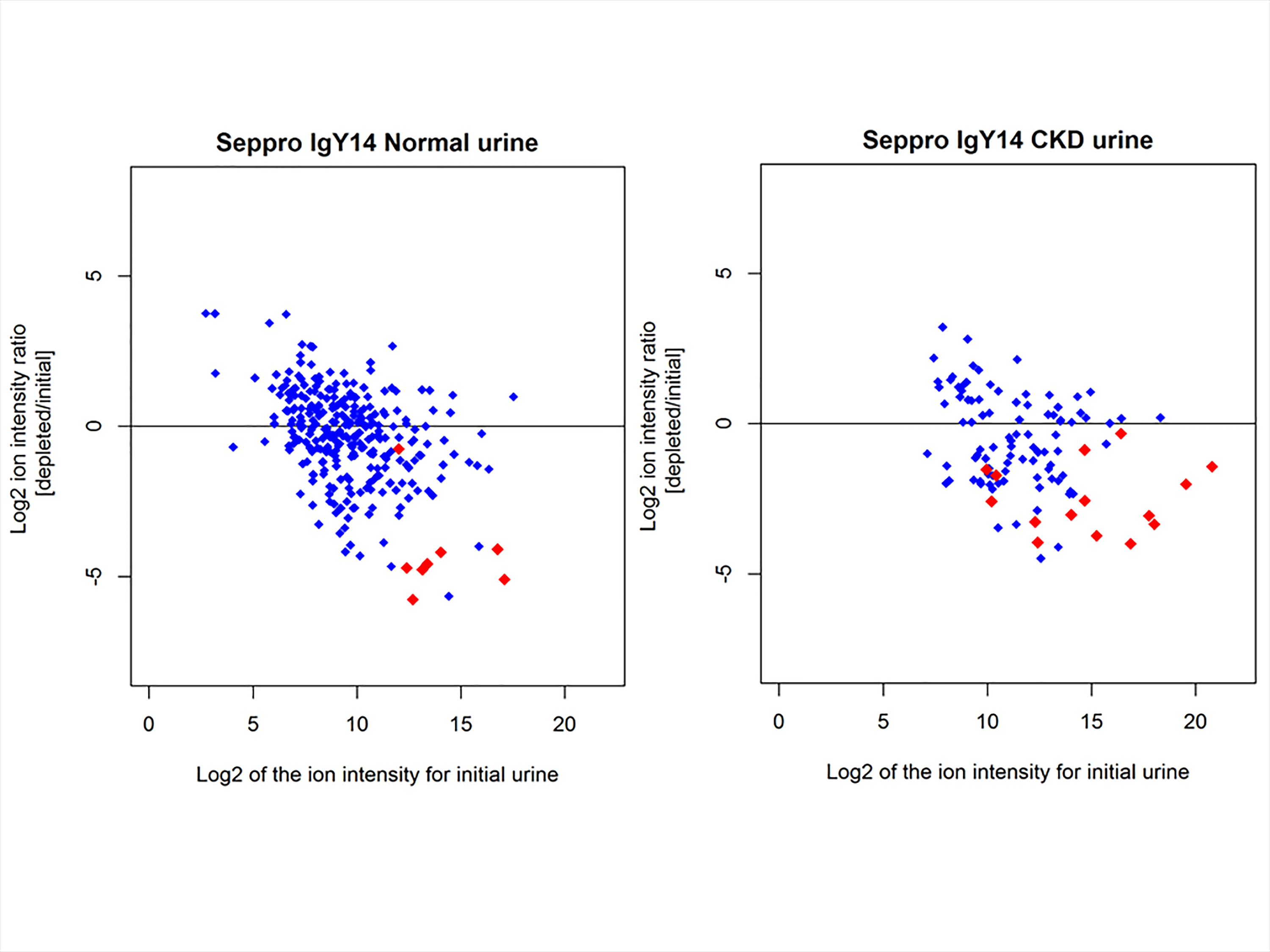

Supplement: S11 Fig — The scatterplots present the protein relative abundance changes after protein depletion in comparison to the initial sample. X axis represents the normalized protein abundance for initial urine in logarithmic scale (log2). Proteins on the Y axis (log2 scale) above a ratio of 0 are enriched in comparison to initial urine, while those below the ratio of 0 are depleted. Proteins with a ratio 0 show the same relative abundance in the initial and depleted sample. Protein abundance for immunoglobulins is presented as a sum of the abundance for all combined proteins in the group. Protein targets for depletion kit are marked as red dots (see Table 1). (TIF) [file pone.0133773.s014.TIF]

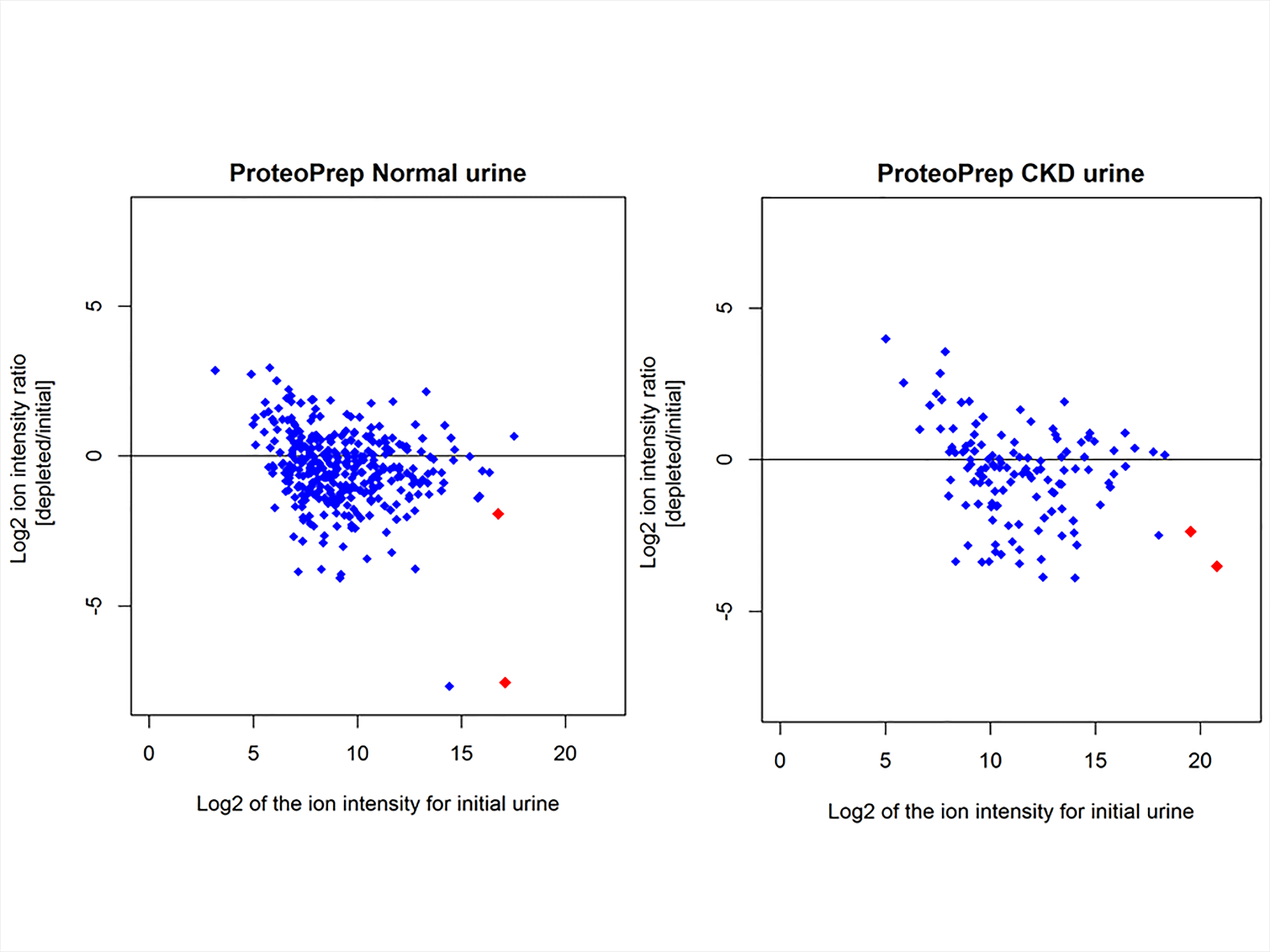

Supplement: S12 Fig — The scatterplots present the protein relative abundance changes after protein depletion in comparison to the initial sample. X axis represents the normalized protein abundance for initial urine in logarithmic scale (log2). Proteins on the Y axis (log2 scale) above a ratio of 0 are enriched in comparison to initial urine, while those below the ratio of 0 are depleted. Proteins with a ratio 0 show the same relative abundance in the initial and depleted sample. Protein abundance for immunoglobulins is presented as a sum of the abundance for all combined proteins in the group. Protein targets for depletion kit are marked as red dots (see Table 1). (TIF) [file pone.0133773.s015.TIF]

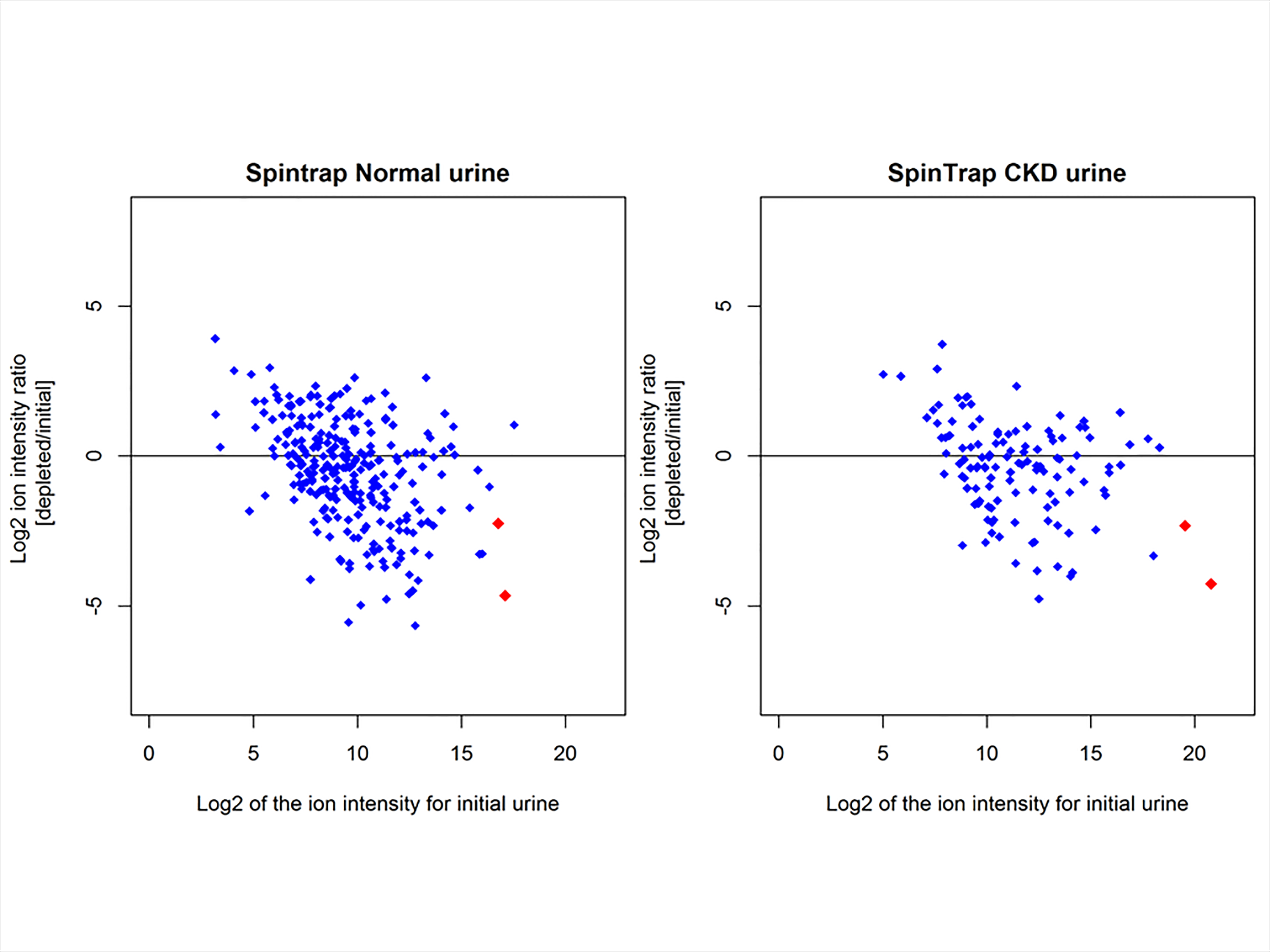

Supplement: S13 Fig — The scatterplots present the protein relative abundance changes after protein depletion in comparison to the initial sample. X axis represents the normalized protein abundance for initial urine in logarithmic scale (log2). Proteins on the Y axis (log2 scale) above a ratio of 0 are enriched in comparison to initial urine, while those below the ratio of 0 are depleted. Proteins with a ratio 0 show the same relative abundance in the initial and depleted sample. Protein abundance for immunoglobulins is presented as a sum of the abundance for all combined proteins in the group. Protein targets for depletion kit are marked as red dots (see Table 1). (TIF) [file pone.0133773.s016.TIF]

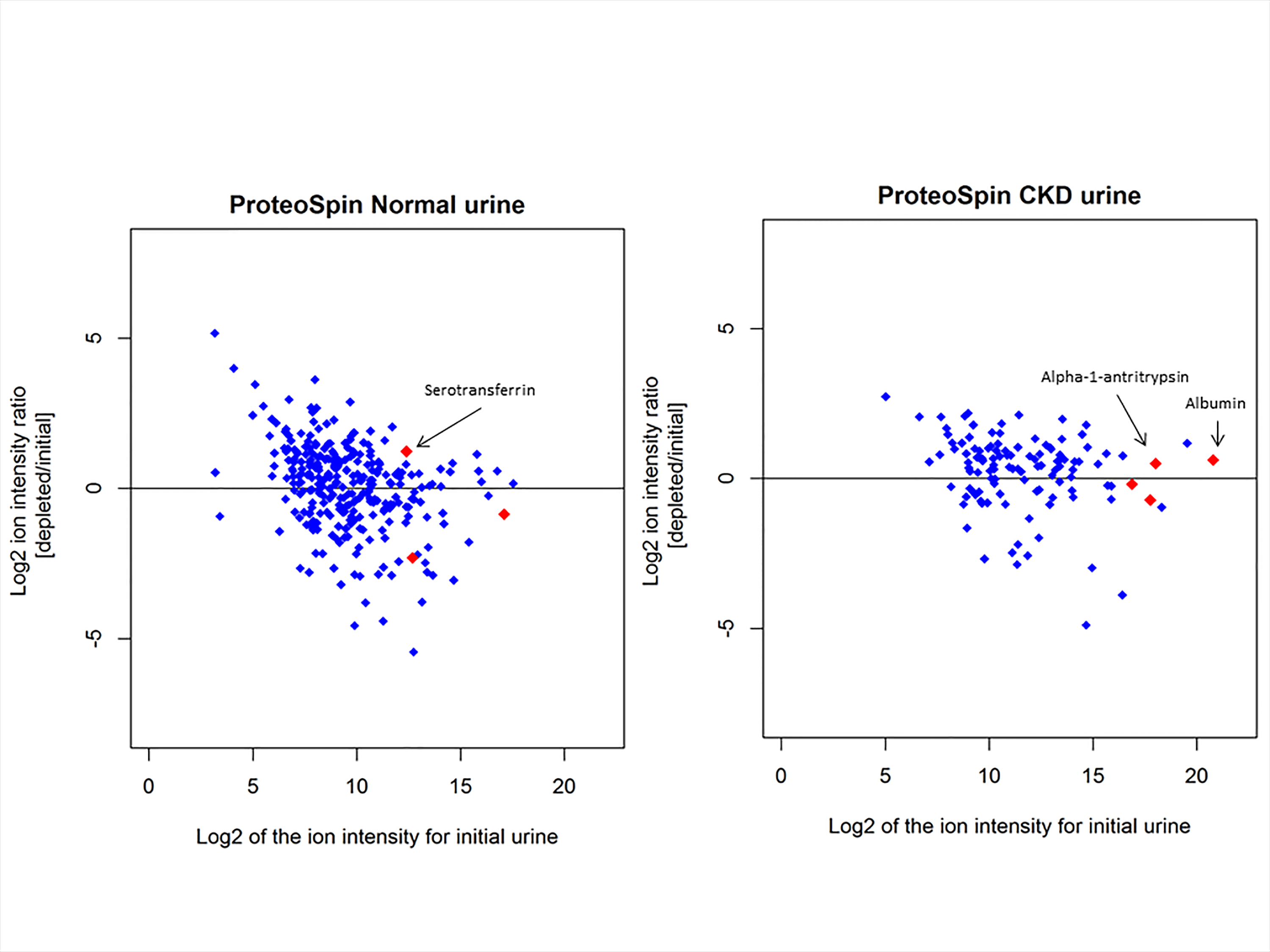

Supplement: S14 Fig — The scatterplots present the protein relative abundance changes after protein depletion in comparison to the initial sample. X axis represents the normalized protein abundance for initial urine in logarithmic scale (log2). Proteins on the Y axis (log2 scale) above a ratio of 0 are enriched in comparison to initial urine, while those below the ratio of 0 are depleted. Proteins with a ratio 0 show the same relative abundance in the initial and depleted sample. Protein abundance for immunoglobulins is presented as a sum of the abundance for all combined proteins in the group. Protein targets for depletion kit are marked as red dots (see Table 1). Protein targets for which the relative abundance increased after depletion are marked by an arrow. (TIF) [file pone.0133773.s017.TIF]
